# U.S. national water and energy land dataset for integrated multisector dynamics research

**DOI:** 10.1038/s41597-022-01290-w

**Published:** 2022-04-20

**Authors:** Jillian Sturtevant, Ryan A. McManamay, Christopher R. DeRolph

**Affiliations:** 1 Department of Environmental Science, Baylor University, Waco, TX 76798 USA; 2 Environmental Sciences Division, Oak Ridge National Laboratory, Oak Ridge, TN 37831 USA

**Keywords:** Environmental impact, Power stations

## Abstract

Understanding resource demands and tradeoffs among energy, water, and land socioeconomic sectors requires an explicit consideration of spatial scale. However, incorporation of land dynamics within the energy-water nexus has been limited due inconsistent spatial units of observation from disparate data sources. Herein we describe the development of a National Water and Energy Land Dataset (NWELD) for the conterminous United States. NWELD is a 30-m, 86-layer rasterized dataset depicting the land use of mappable components of the United States energy sector life cycles (and related water used for energy), specifically the extraction, development, production, storage, distribution, and operation of eight renewable and non-renewable technologies. Through geospatial processing and programming, the final products were assembled using four different methodologies, each depending upon the nature and availability of raw data sources. For validation, NWELD provided a relatively accurate portrayal of the spatial extent of energy life cycles yet displayed low measures of association  with mainstream land cover and land use datasets, indicating the provision of new land use information for the energy-water nexus.

## Abstract (summary)

**Table:** 

Measurement(s)	land use of renewable and nonrenewable technologies
Technology Type(s)	Esri Arcmap
Sample Characteristic - Environment	terrestrial; • aquatic
Sample Characteristic - Location	United States

## Background & Summary

Multi-sector dynamics is a growing field of research aimed at understanding the vulnerabilities and risks of stressors and shocks to interdependencies converging at the intersection of energy, water, and land (EWL) systems^1^. Through the lens of EWL, MSD research examines natural human-environmental system coupling, measures dependencies, and evaluates tradeoffs in resources amongst the various economic sectors^1^. Considerable attention has been devoted solely to evaluating critical interdependencies or tradeoffs within the energy-water nexus, such as studies on thermoelectric water use^2^, drinking water supply and distribution^3^ and wastewater treatment^4^. Although energy and water are critical components of the economy and society, both elements are highly dependent upon land^5^.

Indeed, consideration of food production in sustainability studies has tied landscapes to the energy-water nexus^6^. Beyond the agricultural sector, however, explicit consideration of spatial scales, particularly land dynamics within the energy-water nexus, has seen far less attention^5^. Incorporation of land dynamics within the energy-water nexus engenders a common spatial scale within multi-sector evaluations, such as quantifying land competition among sectors^7^, evaluating resource burdens of alternative socioeconomic choices by way of life cycle analysis^8–10^, or understanding distal teleconnections imbedded within energy life cycles resources^11^.

Understanding the broader environmental impacts among energy technologies requires translating energy life cycle components into landscape-relevant measures. Specifically, mapping land use of energy technologies is imperative, as it provides an estimation of the environmental footprint of electricity consumption^12^ by breaking down the energy types into their respective life cycle steps; production, processing, power generation, and transmission^7,13^. One challenge and necessity in linking energy and water to the land is differentiating land *cover* from land *use*. Traditionally, land cover mapping has relied on remotely sensed multi-spectral imagery to understand the extent of natural ecosystems and human activities in the landscape as seen by the cover of the Earth’s surface, whereas land use describes the “arrangements, activities, and inputs people undertake within a particular land cover type”^14^.

Although products that represent broad land components of energy sectors (e.g., developed land cover, mining) are available for the conterminous United States, current land use data products are limited in their ability to capture all (or at least the majority) of life cycle components of energy sector relevant to landscape analysis^15^. For instance, the national land use dataset (NLUD) is a 30 m product comprised of 79 anthropogenic land use classes and provides an extensive categorization of sectors for land use mapping; however, layers pertaining to energy life cycles and water sectors are limited^16^. The national wall-to-wall anthropogenic land use trends (NWALT) dataset (60 m) provides 19 classes of anthropogenic land use^17^. Although it lacks the specificity of NLUD, it spans five time periods (1976 to 2012) and contains a critical layer (mining) that portrays a key component of energy life cycles^17^. The national land cover dataset (NLCD) is a high-resolution (30 m) depiction of the land surface cover modeled from Landsat imagery and released every five years between 2001 and 2019^14,18^. NLCD depicts 28 classes of land cover, characterized to show complex land cover change^14,18,19^. While these land cover and land use datasets offer broad classification systems that include elements of energy use, they do not represent the majority of the water and land components of energy production life cycles.

Herein, we describe the development of a National Water and Energy Land Dataset (NWELD) depicting the land use of energy production life cycles and water sources pertaining to energy production in the lower 48 states. Although the NLCD extends to Alaska and Hawaii, our exclusion of these states are due to the lack of other land cover/use rasterized data supporting our analysis, particularly the NLUD and NWALT. The various classes of land use in NWELD are, in part, inspired by missing elements shared by NLUD and NWALT, but are extended to provide an operable template for MSD research. Depending on the availability and accuracy of source information, four methodologies involving geospatial techniques were used to create an 86-layer, 30m-resolution gridded product.

## Methods

### Overview of approach

NWELD’s classification scheme is organized by the energy life cycles of major renewable and non-renewable energy sources: coal, hydropower, natural gas, oil, nuclear, bioenergy, solar, and wind (Table 1). These energy life cycles include the extraction, construction (siting and development), production, storage, distribution, and operation of coal, hydropower, natural gas, oil, nuclear, biomass, and general renewable energy sources^20^; some manufacturing is included where it can be linked to an energy production service (i.e., General Renewable Metal Processing Plants). However, NWELD does not include the material acquisition aspects of construction and also excludes decommissioning phases^21^. This is due to the lack of decommissioning geospatial data from OSM and the inability to allocate upstream goods and services to a single energy production source. The inclusion of energy-water life cycles in NWELD are based on relatively simple criteria: the life cycle must have a spatial or mappable component supported by available data or reliable estimation methods. Only life cycles that occur on the surface of the earth are depicted; for example, only surface mines are represented, not underground mines or fields. Aside from extraction of essential fuels or elements that can be strictly tied to a sub-category, sourcing of other raw materials and manufacturing is not included.

**Table 1:** Classes and subclasses of energy life cycles within the NWELD (National Water and Energy Land Dataset) product.

**1. Coal**	**6. Nuclear**	**10. General Renewable Metal Processing Plants**	70. Sunflower	80. Substations
16. Surface Coal Mines	34. Uranium Mines	52. Zinc Metal Processing Plant	12ab. Biodiesel Refinery	81. Transmission Lines
17. Coal Fired Power Plants	35. Uranium In-situ Leaching Plant	53. Silver Metal Processing Plant	**12b. Ethanol**	**14. Infrastructure**
**2. Hydropower**	36. Uranium Mills and Heap Leach Facilities	54. Nickel Metal Processing Plant	12bb. Crops for ethanol	82. Railroad Tracks
18. Hydro Dams	37. Nuclear Power Plant	55. Magnesium Metal Processing Plant	71. Corn	83. Roads, Primary and Secondary
19. Hydro Power Plants	**7. Solar**	56. Lead Metal Processing Plant	72. Barley	84. Flood Control Dams
20. Hydro Dam/Power Plant	38. Quartz Mine for Solar Panels	57. Iron Metal Processing Plant	73. Rice Straw	85. Irrigation Dams
21. Hydro Power Reservoirs	39. Cadmium Mine for Solar Panels	58. Gold Metal Processing Plant	74. Sorghum	86. Navigation Dams
**3. Natural Gas/Oil**	40. Gallium Mine for Solar Panels	59. Copper Metal Processing Plant	75. Sugarcane Bagasse	87. Water Supply Dams
22. Oil and Gas Wells	41. Germanium Mine for Solar Panels	60. Cobalt Metal Processing Plant	76. Switch grass	88. Recreation Dams
23. Hydrocarbon Gas Liquid Pipeline	42. Tellurium Mine for Solar Panels	**11. Mines for Lithium Ion** **Batteries (Renewables)**	12bc. Ethanol refineries	89. Multi-Use Dams
24. Natural Gas/Petroleum Plant	43. Solar Farms	61. Cobalt Mine	**12c. Municipal Landfills with Gas**	**15. Water Sources**
**4. Natural Gas**	**8. Wind**	62. Lithium Mine	**12d. Landfills with Waste and Gas**	90. Water bodies
25. Natural Gas Processing Plant	44. Iron Mine for Windmills	63. Nickel Mine	**12e. Municipal Solid waste**	91. Navigable Rivers
26. Natural Gas Storage Facilities	45.Wind Farms	64. Manganese Mine	12dd. Municipal Landfills	92. Small Network Rivers
27. Natural Gas Power Plant	**9. General Renewable Mines**	**12. Biomass**	12de. Municipal Waste Plant	93. Ocean
28. Natural Gas Pipelines	46. Aluminum Metal Mine	**12a. Biodiesel**	**12f. Woody Biomass**	94. Wastewater Treatment Plant
**5. Oil**	47. Copper Metal Mine	12aa. Crops for Biodiesel	12ee. Woody Solids	
29. Petroleum Refinery	48. Gold Metal Mine	65. Soybean	77. Mills	
30. DOE Petroleum Reserves	49. Silver Metal Mine	66. Rapeseed	78. Forests	
31. Petroleum Power Plant	50. Zinc Metal Mine	67. Canola	79. Both Mills and Forests	
32. Crude Oil Pipelines	51. Lead Metal Mine	68. Mustard	12ef. Wood Waste Plant	
33. Petroleum Pipelines		69. Safflower	**13. Transmission**	

NWELD datasets for each life cycle follow the numeric coding system.

Based on the types of raw datasets available for mapping energy and water features, we conceptualized two general paths for developing land-use models, where they could be used interchangeably: 1) a downscaling model process whereby a combination of rasterized land-cover products (i.e. surrogates) are used in conjunction with information at coarser boundaries to approximate land use for energy in a given area, or 2) direct allocation of land use based on proximate, highly accurate, and mutually exclusive vector boundaries that necessarily identify land use. Part of the problem with the second path lies with the completeness of open-access sources and technological limits to harness those sources that provide high spatial fidelity and accuracy. Developing energy and water land sector mapping is difficult and data intensive; it requires high-resolution depictions of power plants, transmission, and detailed infrastructure in a heavy data format. An example of an accessible data source that provides these types of footprints is Open Street Map (OSM)^14,22^. OSM has become a very popular resource since it is user friendly and created by contributors that describe the attributes of the objects created^14,23,24^. The database provides both local and worldwide vectors^24^ and is edited and improved by thousands of registered users so that their edits are stored and made available for others^25^. OSM provides a valuable resource for developing land use maps of energy and water infrastructures.

**Fig. 1:**
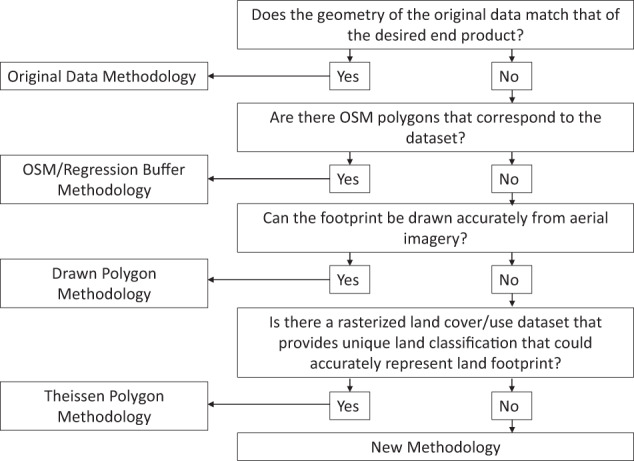
Workflow for developing NWELD based on availability of source information relative to desired end product.

Four methodologies were used depending upon the availability, geometry, granularity, and type of raw data used to represent each energy life cycle (Fig. 1, Supplementary Table 1). Essentially, methodologies  vary in their approach to obtaining geographic boundaries of energy and water infrastructure (via direct observation or estimation) and in how those layers are converted those into a consistent rasterized data product. Geographic boundaries included point data associated with polygon data including OSM polygon footprints, approximations of spatial footprints estimated from models, or manually digitized polygons. Grid cells falling within these boundaries were then reclassified using a refining schema involving previous land sector products, NWALT and NLCD, or directly obtaining pixels from NLCD and reclassifying them according to the various classes. More detailed explanations of the main methodologies are provided in the sections that follow. Detailed stepwise methods used for each layer are provided in Supplementary File 1. All spatial procedures were conducted in ArcMap 10.5, whereas OSM data retrieval was conducted in the R programming environment.

Data products used to develop each of the land use layers are also provided in Supplementary Table 1. Detailed information on raw data used in our analysis, in conjunction with method typologies, are provided in Supplementary Table 2 and Supplementary File 1. Of the data products we utilized, Open Street Map (OSM) was a critical resource to our project. Not only does OSM supply highly resolved polygon data that accurately represents footprints of a given energy and water life cycle, but also the OSM data provides a dataset for developing predictive models of the geographical footprints of energy and water uses in locations where detailed polygon data was unavailable. In the latter case, we used OSM to develop regression equations that estimate the area of buffers based on a set of relevant predictor variables (e.g., Megawatt capacity, structure height). We iteratively obtained OSM polygons for the conterminous US using state names and other search terms within the OSM package^26^ the R programming environment. Search terms varied depending upon the subclass in question. For example, search terms for coal power plants, gas power plants, hydro power plants, nuclear power plants, oil power plants, and solar farms were structured after that of Dunnet *et al*.^22^. Examples of R code for various states and subclasses are provided in the SI text.

### Method types

#### Original data

In cases where the geographic boundaries of a given energy life cycle or water footprint were readily available and accurately represented the final geometry (e.g., roads, railroads, and transmission lines), we extracted rasterized land cover within those known geographic boundaries. This methodology was commonly used for datasets with polygon or line geometry, but also occasionally used with point data if underlying infrastructure had a very small footprint (e.g., natural gas wells) (Supplementary File 1). Polyline and point data were used to extract NLCD raster pixels to convert geometries to a 30 m final product. Depending on the desired end product, the extracted NLCD pixels were reclassified to attain final energy life cycle classes (e.g., wastewater treatment facility polygons were reclassified into “facilities” and “water”). In some cases, source data represented infrastructure with varying widths (ex. roads and transmission lines). In these instances, data was separated according to a particular size class (e.g., transmission voltage category, primary vs secondary roads). To determine widths for each size class, a subset (n = 40) of features for each class were selected and aerial imagery was used to manually record the width of land surrounding each line, which was then averaged for the entire subset and used to create buffers. Buffered lines were then used to extract NLCD raster pixels, representing the infrastructure.

#### OSM/Regression buffer

**Fig. 2:**
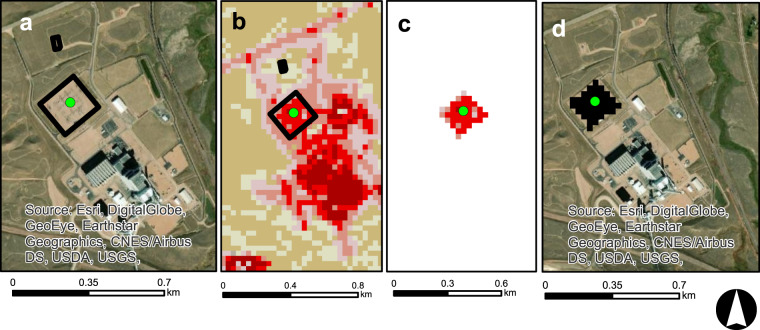
An example of an OSM stepwise process. (**a**) OSM polygons representing a given energy life cycle are obtained using R programming script, (**b**) OSM polygons are associated with other source data (example: green data point) to build regression equations estimating spatial footprints of a given life cycle based on attributes of an energy asset. (**c**) Once spatial footprints are identified, NLCD rasters are extracted and then, (**d**) reclassified to represent the final end product.

**Fig. 3:**
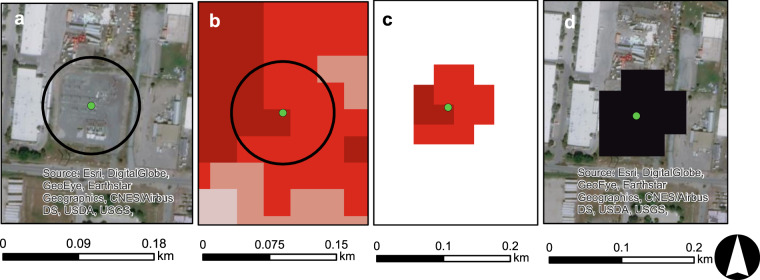
An example of a Regression Buffer stepwise process. (**a**) Point data representing a given energy life cycle are obtained (in this case, a substation). OSM polygons are associated with other source data (example: green data point) to build regression equations estimating spatial footprints of a given life cycle based on attributes of an energy asset. (**b**) The regression equation is applied to produce a buffer (**c**) Once the buffer appropriately encompasses the energy life cycle footprint, NLCD rasters are extracted and then, (**d**) reclassified to represent the final end product.

The second methodology used OSM polygons in two main ways. First, OSM polygons, when available and accurate, were used to directly allocate all land surfaces falling within the geographic boundary of a given energy life cycle at a location (e.g., Fig. 2). In situations where OSM polygons were unavailable but point locations of a given energy life cycle were available, we used predictive models to estimate buffer sizes, which were then applied to points (e.g., Fig. 3). NLCD rasters were then extracted under each buffered area and modified to attain the desired geometry. Using subsets of data where OSM polygons could be paired with ancillary data from other sources, we developed linear regressions to estimate the footprint of an energy life cycle at a given location based on attributes of the location. As one example, OSM polygons were unavailable for the spatial footprint of many hydropower facilities. For instances where OSM polygons provided a footprint of the dam and powerplant, we calculated the OSM area and then paired that information with data on each facility, such as dam height and generating capacity. These variables were then used in regressions to estimate total area of the spatial footprint and were applied to estimate the spatial footprint of points lacking OSM polygons. Total areas were then converted into buffer radii (r) using the circular area equation (*A* = *πr*^2^). Instances of missing OSM data are summarized in Supporting Information 2. Additionally, regression equations for buffer radii and any buffer modifications for energy life cycles are provided in Supporting Information 2.

#### Theissen polygons

**Fig. 4:**
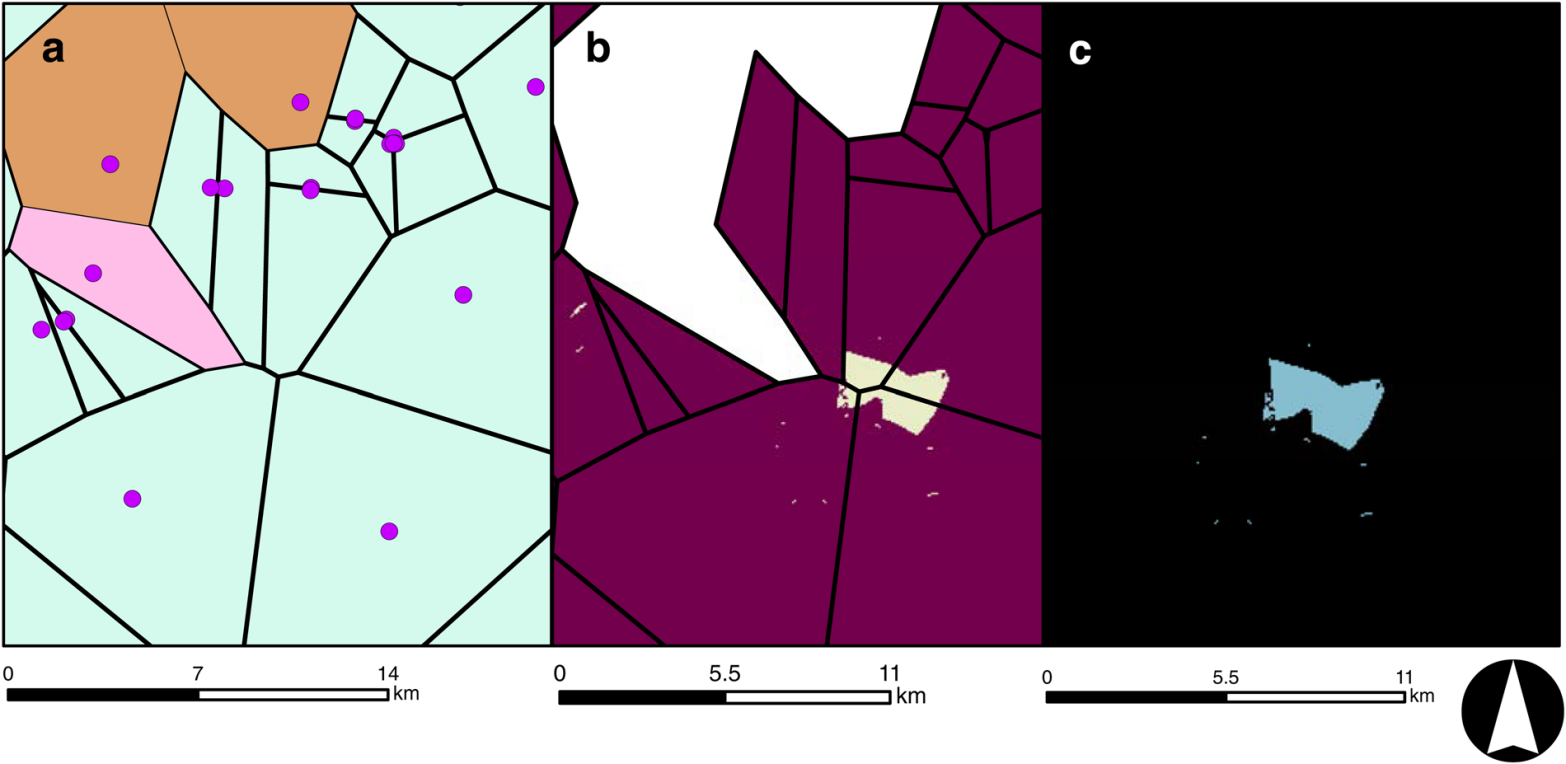
An example of a Theissen Polygon stepwise process (in this case, coal mining) where (**a**) point data representing a given energy life cycle are obtained, (**b**) Theissen polygons are created using ESRI ArcGIS and polygons are separated based on mine classification (**c**) Once spatial footprints are identified, NWALT rasters are extracted and reclassified to represent the final end product.

In cases where regional segregation was required to differentiate among similar land use types (e.g., different types of mining activity), theissen polygons^27^ were used to generate polygon boundaries around clusters of points representing similar land uses (e.g., Fig. 4). This case was particularly important when land use for an energy life cycle was regionally distributed (e.g., mining) and not isolated to patchy instances of land use around individual entities (e.g., power plant). For instance, mining is generally conducted through an entire region, although mines used for different purposes (e.g., coal, minerals) may be clustered in the same area (Fig. 4). Therefore, we could not generally assume that “mining” land classification directly translated into a given energy life cycle simply using regional deduction of resource-rich geology (i.e., coal beds). In these cases, we used theissen polygons to distinguish proximal regions by building polygonal boundaries around clusters of data points (e.g. mines used for same purpose), which represented different energy uses. Rasters underlying each polygon sub-region were then extracted to obtain the desired land product (see Supplementary File 1 for more details).

#### Manually digitized polygons

**Fig. 5:**
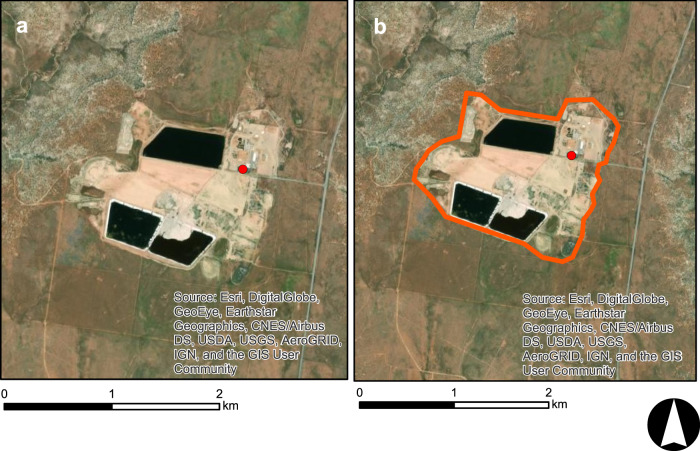
Manually Digitized Polygons stepwise process. (**a**) Point data representing a given energy life cycle are obtained, (**b**) Polygons are developed using ARCGIS tools and aerial imagery as a reference.

Finally, there were situations in which required datasets were either unavailable or lacked the specificity to utilize the previous three methodologies. This generally arose if source datasets had very few observations or there was a complete lack of OSM polygons for a life cycle component. In these cases, we manually digitized spatial footprints using aerial imagery, after which subsequent raster processing followed one of the first two methods (e.g., Fig. 5). For some datasets, sufficient point observations were available and were randomly sampled for manually digitizing footprints. From these samples, linear regressions could be developed and used to estimate buffer radii (as in the second methodology).

## Data Records

**Fig. 6:**
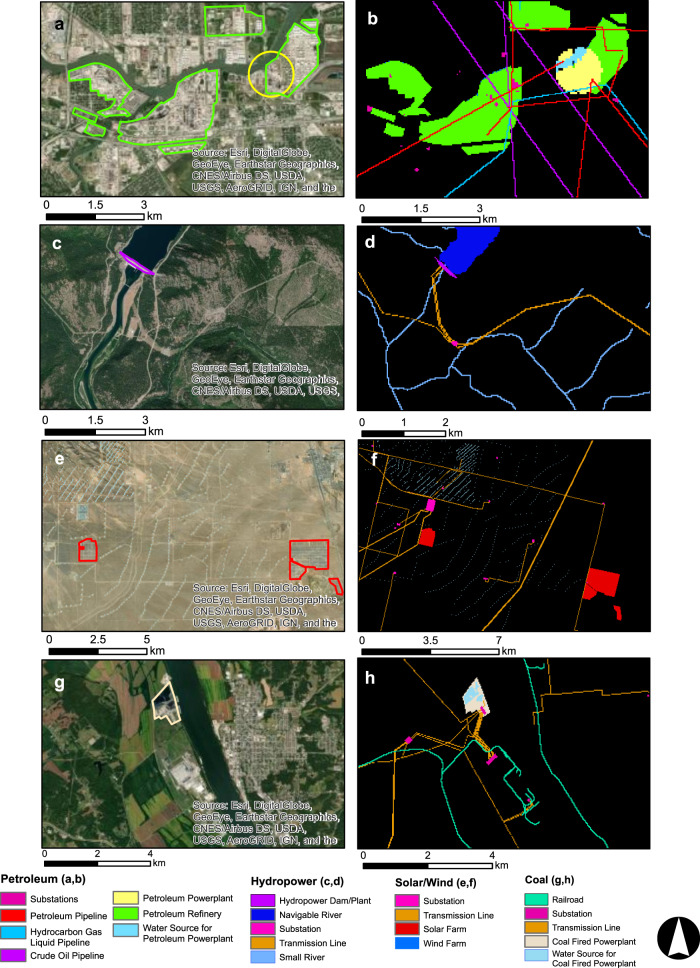
Examples of contextual aerial imagery (left) and the representation of NWELD features (right). (**a**,**b**) petroleum powerplant, refineries, water sources, and associated infrastructure, (**c**,**d**) hydropower dam, power plant, and waterbodies, (**e**,**f**) solar and wind farms and associated infrastructure, (**g**,**h**) coal fired powerplant and associated infrastructures.

Each step of the energy life cycle is a sub-category in the National Water and Energy Land Dataset or NWELD (Table 1). NWELD is a 5-GB (1.32-GB compressed size), 30 m rasterized dataset that can be obtained through figshare^28^. The data is organized according to energy production and water source type, where each layer (surface coal mines, nickel mines, etc.) has an associated gridded integer raster product projected within the North American Datum 83 Conus Albers coordinate system. In total, NWELD has 86 layers for coal, hydropower, natural gas, petroleum, solar, wind, biomass, general renewable, infrastructure, and water sectors as they apply to energy production (Table 1). The layers in each general section (coal, hydropower, etc.) are numbered sequentially to accurately represent the flow of the energy life cycle according to each producer (Table 1). As an example, a series of samples of these sub-layers displayed in Fig. 6 to show the spatial fidelity and differentiation among footprints between the various stages of energy production.

## Technical Validation

The validation of our data product is comprised in four main ways. First, we evaluate the explanatory power (i.e., accuracy) of buffer radii linear regressions used to estimate spatial footprints – this information can be used in future evaluations of spatial footprint assessments in addition to examining potential sources of error in NWELD. Second, we compare NWELD classes to those of NLCD, NWALT and NLUD to differentiate our depiction of energy life cycles from standard land cover and land use products. Third, we used a random stratification process to spot check instances of each energy life cycle layer to determine how well the final product accurately depicts the observed energy use in aerial images. Finally, we compared the aerial footprint of energy technologies in NWELD to comprehensive reviews conducted by Fthenakis and Kim^29^ and Jordaan *et al*.^13^. Applicable life cycles were compared in terms of total area and land use intensity for each technology. A summary of validation results is provided here; however, for the full details of our technical validation, please refer to Supplementary File 3.

The explanatory power of linear regressions for buffer radii varied widely according to energy life cycles; the majority of linear regressions had R^2^ < 0.5. Models with higher performance included natural gas storage facilities (R^2^ = 0.61), solar farms (R^2^ = 0.62), and flood control dams (R^2^ = 0.55). Examples of layers with moderate explanatory power include petroleum refineries (R^2^ = 0.22), several of the layers depicting dams used for various purposes (R^2^ = 0.24–0.46), and substations (R^2^ = 0.39).

**Fig. 7:**
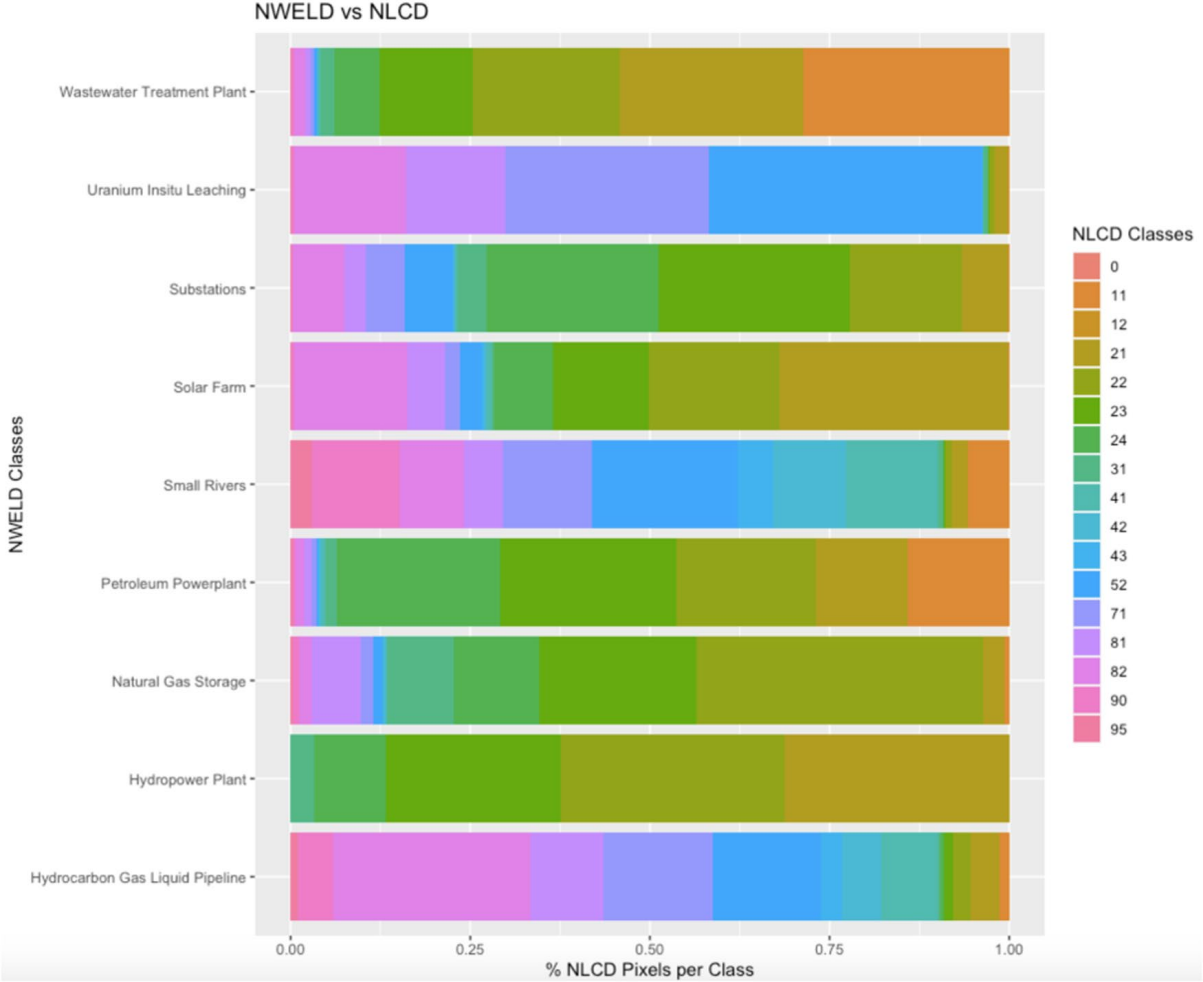
Associations among NWELD layers (y axis) and the NLCD (National Land Cover Dataset) layers (x axis) as a measure of the percent of NLCD pixels associated with a given NWELD layer.

**Fig. 8:**
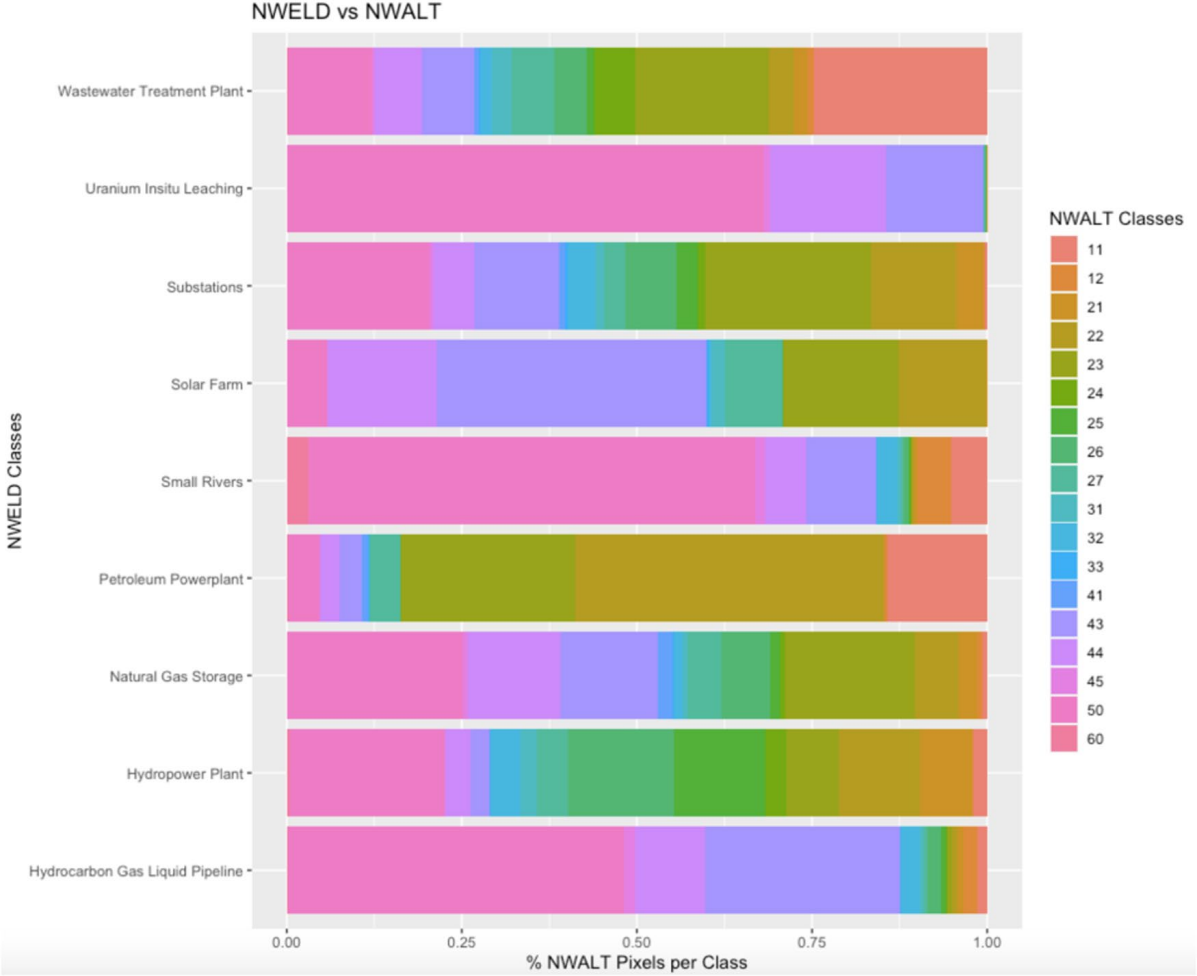
Associations among NWELD layers (y axis) and NWALT (National Wall-to-Wall Anthropogenic Land Use Trends) dataset layers (x axis) as a measure of the percent of NWALT pixels associated with a given NWELD layer.

**Fig. 9:**
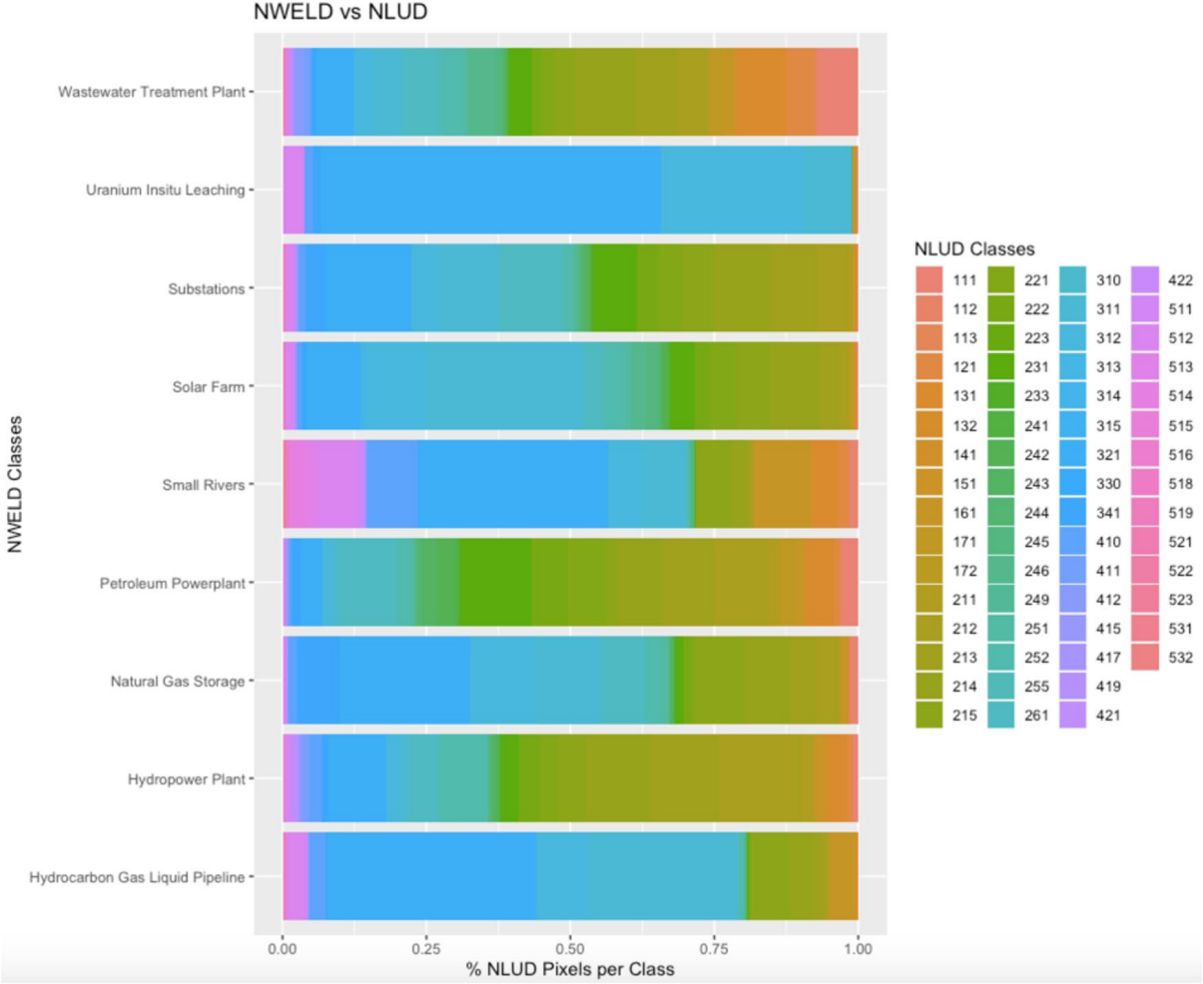
Associations among NWELD layers (y axis) and NLUD (National Land Use Dataset) layers (x axis) as a measure of the percent of NLUD pixels associated with a given NWELD layer.

On a pixel-to-pixel basis, NWELD was compared to NLCD, NWALT, and NLUD via Cramer’s V analysis. This specific analysis was chosen to quantify how much NWELD explains the variability of the other rasterized land use models, or, alternatively, the degree of association between NWELD and NLCD, NWALT, and NLUD. Cramer’s V is represented on a scale of 0 to 1 representing the strength of association between two variables, where 0 indicates no association and 1 is perfect association^30^. Our results indicate that NWELD has little association with previous land use and land cover datasets. Cramer’s V values were 0.210, 0.203, and 0.174 for comparisons between NWELD with NLCD, NWALT, and NLUD, respectively. A visual depiction of this discontinuity between NWELD and contemporary land use-land cover datasets can be seen for nine of the NWELD layers (Figs. 7–9). These cross-comparisons show the level of differentiation that NWELD layers provide relative to the other land classification schemes.

**Fig. 10:**
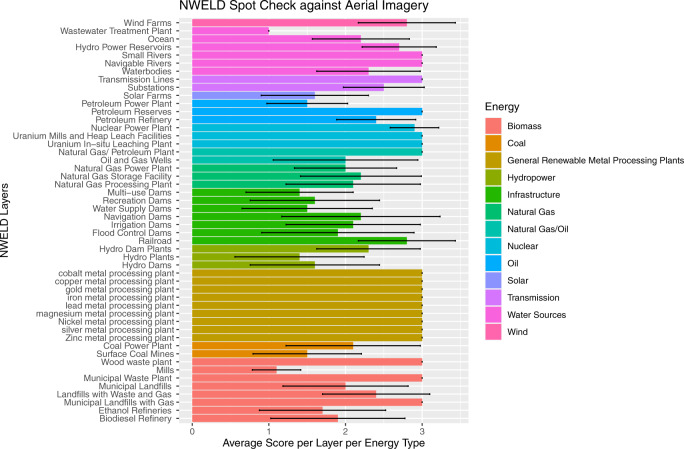
Accuracy assessment of NWELD layers by comparing rasterized products to aerial imagery. Accuracy is measured on a scale of 1 to 3, where a score of 1 indicates that NWELD poorly represents the energy use whereas a score of 3 conveys that NWELD represents the energy use exceedingly well.

Third, as a measure of overall accuracy, NWELD layers were spot checked against aerial imagery and ranked on how well they represented a specified energy use. We used a 4-km grid of the US as a template for randomizing the selection of spatially heterogenous areas for our accuracy assessment. For each NWELD layer, a subset of 10 grids were selected (only where that layer was present) to conduct a visual comparison of the accuracy in the raster layer’s representation of edges and extent of a given energy life cycle compared to aerial imagery (e.g., power plant, surface mine, reservoir, dam). Each sample was assigned a score from 1 to 3, indicating poor to excellent representation, respectively. Average scores are reported by layer (Fig. 10) and by energy production type (transmission, biomass, solar, etc.) (Supplementary File 3, Table S8).

General renewable metal processing plants are very well represented by NWELD and ranked highest. Nuclear was the second best represented (2.97), followed by wind (2.8), transmission (2.75), natural gas/oil (2.5), water sources (2.37), oil (2.3), biomass (2.26), natural gas (2.1), infrastructure (1.93), coal (1.8), hydropower (1.77), and solar (1.6). The above process included 50 of the 86 NWELD layers. The remaining layers, such as underground mines/pipes, biomass crops, and biomass forests/mills were excluded because they were unsuitable for aerial assessment (i.e., aerial information could not reliably identify these features).

**Fig. 11:**
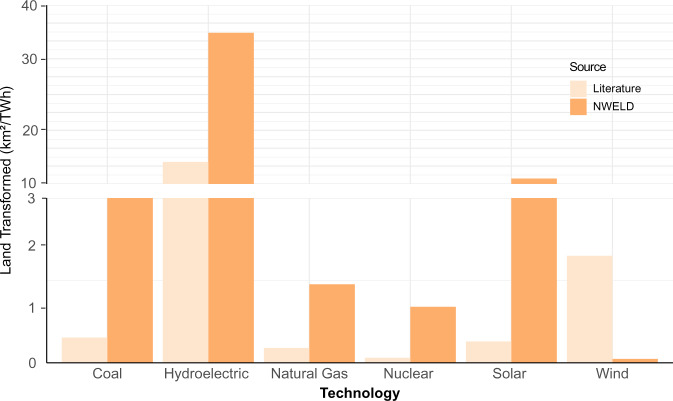
Comparison of area(km^2^) per Terawatt-hour between applicable NWELD layers and calculations from the literature^13,29^. The following graph portrays the amount of land transformed from renewable energy production technologies such as hydropower, solar, and wind and non-renewable production technologies such as coal, natural gas, and nuclear.

Finally, to examine the robustness of our geospatial methods, we compared applicable NWELD layers to the mathematical calculations of previous reviews of land used for electricity^13,29^ (Fig. 11). First, we cross referenced NWELD to each review and chose land uses that were directly comparable. Following this selection method, we assessed the following energy technologies and determined their area (km^2^) per Terawatt-hour: coal, natural gas, nuclear, hydropower, wind, and solar. To calculate land intensity values for NWELD, we obtained electricity consumption estimates for the conterminous US from the Energy Information Administration (https://www.eia.gov/totalenergy/data/annual/). See Supplementary File 3 for more details on calculations.

In terms of land transformation (km^2^ TWh^−1^), NWELD’s estimates are larger than the literature, with wind as the exception (Fig. 11). The literature calculations depict coal (0.46), natural gas (0.27), solar (0.39), and nuclear (0.09), as being lower than two renewable technologies - hydroelectric (13.68) and wind (1.95). NWELD reflects a similar trend, in which coal (3.92), natural gas (1.42), nuclear (1.02), and wind (0.07) are lower than the renewable technologies hydroelectric (35.38) and solar (10.89). Generally, hydroelectric technology has the largest land transformation from both sources. According to NWELD, wind has the lowest land transformation, whereasnuclear has the lowest land transformation according to the literature.

## Usage Notes

NWELD offers a rasterized dataset depicting the land use of energy resource extraction, transportation, production, and operations, as well as the land use of water sources related to energy production. Each raster layer is provided at 30-m resolution and depicts the spatial distribution of a respective energy life cycle category, where the spatial extent only maps a life cycle’s presence, denoted as 1. Raster layers can be summarized using a number of spatial statistics, such as agglomerating grid results to coarser regions (e.g., zonal statistics, area tabulation using ESRI ArcMap or Raster package^31^ in the R programming environment. Additionally, by way of map algebra (ESRI) or raster math (raster package, R), NWELD’s raster surfaces can be translated into measures of flux or risk surfaces.

NWELD is a dramatic improvement in understanding land use for energy production and transmission, whereas previous land use/cover rasterized datasets such as NLCD, NWALT, and NLUD provide rather vague land uses/covers that do not differentiate amongst these detailed uses. The granularity and specificity of land use data afforded by NWELD could be used to compare and validate previous spatial footprint calculation methods used to compare energy technologies^32,33^. Additionally, NWELD could be helpful in updating power density models^32^, more accurately calculating greenhouse gas (GHG) emissions^21^, studying the energy sector’s socioeconomic impacts^33^, or investigating the most compatible energy types for specific urban areas^34^. NWELD also provides a resource for land use planning. Specifically, land management organizations can utilize NWELD to create innovative land use plans that consider the effect of energy consumption, production, and resource extraction on local ecosystem integrity and habitat fragmentation. NWELD provides a means to explore regional heterogeneity in the land-use efficiencies of energy technologies to cross-compare energy resource assessments with land consumption and minimize future deployment impacts by avoiding regions where land-use efficiency is low.

Among the limitations of NWELD are that it approximates area coverage. Therefore, NWELD is not intended to be used as a site-by-site assessment of the precise layout of the US’s energy infrastructure, but rather, a spatial product for regional assessment, modeling, and nexus applications. Approximations of the energy life cycle footprints are based on the layer’s methodology, underlying datasets, including the accuracy of rasterized datasets used to refine the final product. As indicated, we used linear regressions to estimate the area of polygon buffers, i.e., the footprint of a given energy life cycle. While this is undoubtedly a source of error, these models could also be useful for predicting land area requirements or projecting future land use development related to energy resource deployment.

## Supplementary information

Supplementary File 2

Supplementary File 3

Supplementary Table 1

Supplementary Table 2

Supplementary File 1

## Data Availability

Geospatial processing was primarily conducted using pre-existing tools within ESRI ArcMap 10.5. However, data retrieval, specifically OSM data, was obtained using code within the OSM package of the R programming environment. Technically, the code primarily utilized pre-existing routine function calls supported within the OSM library, specifically determining bounding geographies (in our case, US states) for OSM feature retrieval and using search terms for “keys” (major groups of objects) and “values” (sub-classifications of keys). Keys and values were varied and dependent upon each energy life cycle. Reproducible examples of code are provided in Supplementary File 2, Tables S2-3.
