# Supplementary material for: U.S. national water and energy land dataset for integrated multisector dynamics research
Source: Sci Data. 2022 Apr 20;9:183. doi: 10.1038/s41597-022-01290-w (PMC9021314; doi:10.1038/s41597-022-01290-w)
Supplement: Supplementary file 1 — Supplementary File 2 [file 41597_2022_1290_MOESM1_ESM.docx]

Supplementary File 2.
Methods for Open Street Map Data Searches and Buffer Radii Regression Equations

**U.S. national water and energy land dataset for integrated multisector dynamics research**

Jillian Sturtevant^1^, Ryan A. McManamay^1*^, Christopher R. DeRolph^2^

^1^*Department of Environmental Science, Baylor University, Waco, TX 76798*

^2^Environmental Sciences Division, Oak Ridge National Laboratory, Oak Ridge, TN 37831

*Corresponding Author

[Ryan_McManamay@baylor.edu](mailto:Ryan_McManamay@baylor.edu)

One Bear Place #97622

Waco, Texas 76798-7266

**Table S2-1. List of missing OSM data by state, including Washington D.C. P=plant data, G=generator**

| **State** | **Coal** | **Hydro** | **Nuclear** | **Wind** | **Solar** | **Oil** | **Gas** |
| --- | --- | --- | --- | --- | --- | --- | --- |
| Alabama |  |  |  |  |  | G, P |  |
| Alaska |  |  |  |  |  |  |  |
| Arizona |  |  |  |  |  | G, P |  |
| Arkansas |  |  | P |  |  | G, P |  |
| California |  |  |  |  | G | G |  |
| Colorado |  |  |  |  |  |  |  |
| Connecticut |  |  |  | P | G | P | P |
| Delaware | P |  |  | G, P |  | G, P | P |
| Florida |  | P |  |  |  |  |  |
| Georgia |  |  |  |  |  | G, P |  |
| Hawaii |  | G, P |  |  |  |  |  |
| Idaho | P |  |  |  |  | G, P |  |
| Illinois |  |  |  |  |  | G, P |  |
| Indiana |  | P |  | P |  | G, P |  |
| Iowa |  |  | G |  |  | P |  |
| Kansas |  | G |  |  |  | G, P |  |
| Kentucky |  |  |  |  |  | G, P |  |
| Louisiana |  |  |  |  | P | G, P |  |
| Maine |  |  |  |  | P |  | G |
| Maryland |  |  |  | P |  |  |  |
| Massachusetts |  |  |  | P |  | P |  |
| Michigan |  |  |  |  |  | P |  |
| Minnesota |  |  |  |  |  |  |  |
| Mississippi | P |  |  |  | P | G, P |  |
| Missouri |  |  |  |  |  | G, P |  |
| Montana | P |  |  |  | G, P | G, P | P |
| Nebraska |  |  |  |  |  | G, P |  |
| Nevada |  |  |  |  |  | G, P |  |
| New Hampshire | G |  |  |  |  | P |  |
| New Jersey |  |  |  | P |  | P |  |
| New Mexico |  |  |  |  |  | G, P |  |
| New York | G | P | G, P | P | P | P |  |
| North Carolina |  |  |  | P |  |  |  |
| North Dakota |  | G |  |  |  | G, P | G, P |
| Ohio |  | P |  |  |  | G, P |  |
| Oklahoma |  |  |  |  | G, P | P |  |
| Oregon | G |  |  |  |  |  |  |
| Pennsylvania |  |  |  | P |  | P |  |
| Rhode Island |  | G, P |  | P |  | G, P | P |
| South Carolina |  |  |  |  |  |  |  |
| South Dakota | P |  |  |  | G, P | G, P |  |
| Tennessee |  |  |  | P |  | G, P |  |
| Texas |  |  |  |  |  | P |  |
| Utah |  |  |  |  |  | P |  |
| Vermont |  |  |  |  |  | P |  |
| Virginia |  |  |  |  |  |  |  |
| Washington |  |  |  |  |  |  |  |
| West Virginia |  |  |  | P |  |  |  |
| Wisconsin |  |  |  |  |  | P |  |
| Wyoming |  |  |  |  | G, P | G, P | G, P |
| Washington D.C. | P |  | G, P | G, P | P | G, P | G, P |

**Table S2-2. List of available OSM Data for Coal and Uranium Mines**

| **States with Surface Coal Mines** | **States with Uranium Mines** |
| --- | --- |
| Alabama | Arizona |
| Alaska | California |
| Arizona | Colorado |
| Colorado | Kansas |
| Illinois | Montana |
| Indiana | Nevada |
| Kentucky | New Mexico |
| Louisiana | North Dakota |
| Maryland | South Dakota |
| Montana | Texas |
| New Mexico | Utah |
| North Dakota | Washington |
| Ohio | Wyoming |
| Oklahoma |  |
| Pennsylvania |  |
| Tennessee |  |
| Texas |  |
| Utah |  |
| Virginia |  |
| West Virginia |  |
| Wyoming |  |

**Table S2-3. Example of code produced in R programming Environment using the OSM package**

| **Dams** |
| --- |
| a<- opq(bbox='California, United States', timeout = 100000)  a<- add_osm_feature(a, key='waterway', value = 'dam')  osmdata_xml(a, filename = 'CAdam.osm' ) |
| **Substations** |
| a<- opq(bbox='California, United States', timeout = 100000)  a<- add_osm_feature(a, key='power', value = 'substation')  osmdata_xml(a, filename = 'CAsub.osm' ) |
| **Wastewater Treatment Plants** |
| a<- opq(bbox='California, The United States of America', timeout=100000) %>%      add_osm_feature(key='man_made', value= 'wastewater_plant')  osmdata_xml(a, filename = 'CAwastew.osm' ) |
| **Solar Farms** |
| a<- opq(bbox=’California, United States’, timeout=100000)%>%  a<- add_osm_feature(q, key=’generator:source’,value=’solar’)%>%  osmdata_xml(a, filename=’calsolar.osm’)    b<- opq(bbox=’California, United States’, timeout=100000) %>%  b<- add_osm_feature(q, key=’plant:source’,value=’solar’) %>%  osmdata_xml(b, filename=’calsolar_2.osm’) |
| **Coal Power Plant** |
| a<- opq(bbox=’California, United States’, timeout=100000)%>%  a<- add_osm_feature(q, key=’generator:source’,value=’coal’)%>%  osmdata_xml(a, filename=’calcoal.osm’)    b<- opq(bbox=’California, United States’, timeout=100000) %>%  b<- add_osm_feature(q, key=’plant:source’,value=’coal’) %>%  osmdata_xml(b, filename=’calcoal_2.osm’) |
| **Hydropower Plant** |
| a<- opq(bbox=’California, United States’, timeout=100000)%>%  a<- add_osm_feature(q, key=’generator:source’,value=’hydro’)%>%  osmdata_xml(a, filename=’calhydro.osm’)    b<- opq(bbox=’California, United States’, timeout=100000) %>%  b<- add_osm_feature(q, key=’plant:source’,value=’hydro’) %>%  osmdata_xml(b, filename=’calhydro_2.osm’) |

| **Nuclear Power Plant** |
| --- |
| a<- opq(bbox=’California, United States’, timeout=100000)%>%  a<- add_osm_feature(q, key=’generator:source’,value=’nuclear’)%>%  osmdata_xml(a, filename=’calnuclear.osm’)    b<- opq(bbox=’California, United States’, timeout=100000) %>%  b<- add_osm_feature(q, key=’plant:source’,value=’nuclear’) %>%  osmdata_xml(b, filename=’calnuclear_2.osm’) |
| **Wind Farm** |
| a<- opq(bbox=’California, United States’, timeout=100000)%>%  a<- add_osm_feature(q, key=’generator:source’,value=’wind’)%>%  osmdata_xml(a, filename=’calwind.osm’)    b<- opq(bbox=’California, United States’, timeout=100000) %>%  b<- add_osm_feature(q, key=’plant:source’,value=’wind’) %>%  osmdata_xml(b, filename=’calwind_2.osm’) |
| **Natural Gas Power Plant** |
| a<- opq(bbox=’California, United States’, timeout=100000)%>%  a<- add_osm_feature(q, key=’generator:source’,value=’gas’)%>%  osmdata_xml(a, filename=’calgas.osm’)  b<- opq(bbox=’California, United States’, timeout=100000) %>%  b<- add_osm_feature(q, key=’plant:source’,value=’gas’) %>%  osmdata_xml(b, filename=’calgas_2.osm’) |
| **Oil Storage** |
| e<- opq(bbox='California, United States of America', timeout = 100000)%>%    add_osm_feature(key='man_made', value= 'storage_tank') %>%    add_osm_feature(key = 'content', value= 'oil')  osmdata_xml(e, filename = 'CAOilStorage.osm' ) |
| **Oil Refineries** |
| e<- opq(bbox='California, United States of America', timeout = 100000)%>%    add_osm_feature(key='industrial', value= 'refinery') %>%    add_osm_feature(key = 'refinery', value= 'oil')  osmdata_xml(e, filename = 'CAOilRefine.osm' ) |
| **Natural gas Storage** |
| e<- opq(bbox='California, United States of America', timeout = 100000)%>%    add_osm_feature(key='man_made', value= 'storage_tank') %>%    add_osm_feature(key = 'content', value= 'LNG')  osmdata_xml(e, filename = 'CANGStore.osm' ) |

| **Natural Gas Processing Plants** |
| --- |
| e<- opq(bbox='California, United States of America', timeout = 100000)%>%    add_osm_feature(key='industrial', value= 'refinery') %>%    add_osm_feature(key = 'refinery', value= 'gas')  osmdata_xml(e, filename = 'CANGProcess.osm' ) |
| **Oil Pipelines** |
| e<- opq(bbox='California, The United States of America', timeout=100000) %>%      add_osm_feature(key='man_made', value= 'pipeline') %>%    add_osm_feature(key='substance', value= 'oil') %>%    add_osm_feature(key='location', value= 'overground') %>%    add_osm_feature(key='usage', value= 'transmission')  osmdata_xml(e, filename = 'CA_OilPipe.osm' ) |
| **Natural Gas Pipelines** |
| e<- opq(bbox='California, The United States of America', timeout=100000) %>%    add_osm_feature(key='man_made', value= 'pipeline') %>%    add_osm_feature(key='substance', value= 'gas') %>%    add_osm_feature(key='location', value= 'overground') %>%    add_osm_feature(key='usage', value= 'transmission')  osmdata_xml(e, filename = 'CA_NGPipe.osm' ) |
| **Coal Mines** |
| a<- opq(bbox='Alabama, United States', timeout = 100000)  a<- add_osm_feature(a, key='resource', value = 'coal')  osmdata_xml(a, filename = 'ALcmine.osm' ) |
| **Mineshafts** |
| a<- opq(bbox='Alabama, United States', timeout = 100000)  a<- add_osm_feature(a, key='man_made', value = 'mineshaft')  osmdata_xml(a, filename = 'ALshaft.osm' ) |
| **Uranium Mines** |
| m<- opq(bbox='Colorado, United States', timeout = 100000)  m<- add_osm_feature(m, key='resource', value = 'uranium')  osmdata_xml(m, filename = 'COumine.osm' ) |
| **Landfills** |
| a<- opq(bbox='Alabama, United States', timeout = 100000)  a<- add_osm_feature(a, key='landuse', value = 'landfill')  osmdata_xml(a, filename = 'AL_LF.osm' ) |

**Table S2-4. Regression equations for the layers created via the OSM/Regression Buffer Methodology**

| **Coal Power Plant** |
| --- |
| Area [meter squared] =  (210655.8246+( 20*217233.6442)) + ((501.9526292+(20*164.7155905)) * [Coal Megawatt]) |
| **Ethanol Plant** |
| Area [meter squared] =  (266635.637+( 15*407861.5477)) + (( -38851.36213+(15*142242.4765)) * [PADD])  +((5258.574897+(15*2043.264822)) * [Capacity]) |
| **Biodiesel Plant** |
| Area [meter squared] =  (489130.1546+(10*939085.1775)) + ((24872.12355+(10*306315.6073)) * [PADD])  +((10790.14305+(10*10680.67388)) * [Cap Mm gal])    Mean of OSM polygons designated by point data in the ‘Biodiesel’ subclass:  554282.573829 meters squared |
| **Petroleum Plant** |
| Area [meter squared] =  (1883872.547+( 2.5*685321.5818)) + ((39.27064721 +(2.5*41.46785352)) * [Capacity])  +(( -27.8939881+(2.5*40.92364655)) * [Crude]) |
| **Oil Power Plant** |
| Area [meter squared] =  (32053.76803+( 50*21634.04723)) + ((99.01192833+(50*58.09734978)) * [Crude Megawatt]) |
| **Natural Gas Processing Plant** |
| Area [meter squared] =  (-456862.1659+( 2*1165111.103)) + (( -803.8047502+( 2*1259.899221)) * [Gas cap])  +((1315.990116+( 2*1597.357258)) * [Plant flow])  +((548.8807029+(2*989.2886607)) * [BTU content])    Mean of OSM polygons designated by point data in the ‘Natural Gas Processing Plant’ subclass:  193795.748652 squared meters |

| **Natural Gas Power Plant** |
| --- |
| Area [meter squared] =  (112545.4912+( 40*66366.76796)) + ((219.5715341+(40*99.76464902)) * [Natural gas Megawatt]) |
| **Natural Gas Storage** |
| Area [meter squared] =  (49610.59397+( 78112.6555)) + ((0.792968852+(0.108592972)) * [MAXDEL])  +(( -0.00873776+( 0.003309681)) * [Work cap])    Mean of OSM polygons designated by point data in the ‘Natural Gas Storage’ subclass:  169662.804031 meters squared |
| **Substation** |
| Area [meter squared] =  (-14294.80301+( 3*232.7951284)) + ((2684.866021+( 3*48.83482745)) * [Lines])  + ((106.607044+( 3*1.449075506))* [Max volt])    Mean of OSM polygons designated by point data in the ‘Substation’ subclass:  7952.734014 meters squared |
| **Hydropower Dam** |
| Area [meter squared] =  (4802.89081+(20*2105.95506)) + ((78.69283284+(20*19.78214222)) * [height])  + ((2.382814433+(20*0.245899129)) * [length]) |
| **Hydropower Plant** |
| Area [meter squared] =  (4558.594286+(20*1391.213821)) + (( -0.527907218+(20*15.18740844)) * [Hydropower Megawatt]) |
| **Nuclear Power Plant** |
| Area [meter squared] =  (311980.5835+(25*542400.8142)) + ((301.8432866+(25*311.6485266)) * [Nuclear Megawatt]) |

| **Solar Farm** |
| --- |
| Area [meter squared] =  (1825.948959+( 130*28453.44845)) + ((28264.48559+(130*680.0838604)) * [Solar Megawatt]) |
| **Flood Control Dam** |
| Area [meter squared] =  (-9139.425679+(20*2395.088001)) + ((9.543114274+( 20*0.370826354)) * [Dam length])  +((283.7045692+( 20*54.05298605)) * [Dam height])    Mean of OSM polygons designated by point data in the ‘Flood Control Dam’ subclass:  16457.260043 meter squared |
| **Irrigation Dam** |
| Area [meter squared] =  (485.7102262+( 30*467.3763489)) + ((6.908659381+( 30*0.381030704)) * [Dam length])  +((77.43659804+( 30*11.12195026)) * [Dam height]) |
| **Multi-Use Dam** |
| Area [meter squared] =  (1057.427528+(20* 1452.344203)) + ((7.105107445+(20* 0.353785845)) * [Dam length])  +((113.0195178+( 20*17.10822218)) * [Dam height]) |
| **Navigation Dam** |
| Area [meter squared] =  (10597.74836+(25*2201.639089)) + ((0.554575031+(25* 0.621880733)) * [Dam length])  +((-357.6275961+( 25*523.4337577)) * [Dam height]) |
| **Recreation Dam** |
| Area [meter squared] =  (1321.358833+( 90*212.3130667)) + ((4.568291199+(90* 0.222281282)) * [Dam length])  +((72.20349176+(90*7.213819823)) * [Dam height]) |
| **Water Supply Dam** |
| Area [meter squared] =  (-24301.06581+( 10*11679.12431)) + ((5.010730495+(10*3.787133634)) * [Dam length])  +((930.3538058+(10*192.0612898)) * [Dam height])    Mean of OSM polygons designated by point data in the ‘Water Supply Dam’ subclass:   22170.510636 meters squared |
| **Landfills with Waste and Gas** |
| Area [meter squared] =  (1320246.955+( 1.5*92163.85819)) + (( -68173.21609+( 1.5*134197.0326)) * [Install MW])  +((92469.07622+(1.5*146602.5032)) * [Bio MW])  Area [meter squared] =  (1320246.955+( 3*92163.85819)) + (( -68173.21609+( 3*134197.0326)) * [Install MW])  +((92469.07622+(3*146602.5032)) * [Bio MW]) |
| **Municipal Landfills** |
| Mean of OSM polygons designated by point data in the ‘Municipal Landfills’ subclass:   696283.8 meters squared |
