# Supplementary material for: U.S. national water and energy land dataset for integrated multisector dynamics research
Source: Sci Data. 2022 Apr 20;9:183. doi: 10.1038/s41597-022-01290-w (PMC9021314; doi:10.1038/s41597-022-01290-w)
Supplement: Supplementary file 2 — Supplementary File 3 [file 41597_2022_1290_MOESM2_ESM.docx]

Supplementary File 3.

Detailed Results of Technical Validation for NWELD

**U.S. national water and energy land dataset for integrated multisector dynamics research**

Jillian Sturtevant^1^, Ryan A. McManamay^1*^, Christopher R. DeRolph^2^

^1^*Department of Environmental Science, Baylor University, Waco, TX 76798*

^2^Environmental Sciences Division, Oak Ridge National Laboratory, Oak Ridge, TN 37831

*Corresponding Author

[Ryan_McManamay@baylor.edu](mailto:Ryan_McManamay@baylor.edu)

One Bear Place #97622

Waco, Texas 76798-7266

**Table S3-1. Diagnostic tables of R^2^ error values for OSM polygon regressions.**

| **General Layers Used** | **Life Cycle Sub-Layers** | **R^2^ Value** |
| --- | --- | --- |
| Coal | Coal Fired Power Plants | 0.06436 |
| Hydropower | Hydro Dams | 0.36110 |
|  | Hydropower Plants | 2.4657E-05 |
| Natural Gas | Natural Gas Processing Plant | 0.05068 |
|  | Natural Gas Power Plant | 0.01864 |
|  | Natural Gas Storage Facilities | 0.61367 |
| Oil | Petroleum Refinery | 0.22474 |
|  | Petroleum Power Plant | 0.13259 |
| Nuclear | Nuclear Power Plant | 0.01998 |
| Solar | Solar Farms | 0.61925 |
| Transmission | Substations | 0.39152 |
| Infrastructure | Flood Control Dams | 0.55130 |
|  | Irrigation Dams | 0.46467 |
|  | Navigation Dams | 0.04250 |
|  | Water Supply Dams | 0.04612 |
|  | Recreation Dams | 0.29873 |
|  | Multi-Use Dams | 0.23817 |
| Biomass | Biodiesel Refinery | 0.02847 |
|  | Ethanol Refinery | 0.18778 |
|  | Municipal Landfills with Gas and Waste | 0.00768 |

**Table S3-2. Associations between 9 NWELD samples and the NLCD raster dataset explained by number of NLCD pixels per NWELD layer**

| **NLCD Layers** | **NWELD sample classes (Number of Pixels)** |  | | | | | | | | |
| --- | --- | --- | --- | --- | --- | --- | --- | --- | --- | --- |
|  | Hydrocarbon Gas Liquid Pipeline | Hydropower Plant | Natural Gas Storage | Petroleum Power plant | Small Rivers | Solar Farm | Substations | Uranium Insitu Leaching | Wastewater Treatment Plant |  |
| Barren Land | 5757 | 235 | 2383 | 4987 | 1508883 | 14429 | 16168 | 137 | 40422 |  |
| Cultivated Crops | 500533 | 0 | 465 | 3607 | 19338408 | 665877 | 29039 | 3232 | 33660 |  |
| Deciduous Forest | 144265 | 0 | 66 | 1902 | 28056009 | 27608 | 1764 | 10 | 6103 |  |
| Developed, High Intensity | 9436 | 698 | 3075 | 71742 | 174986 | 346565 | 97636 | 10 | 131039 |  |
| Developed, Low Intensity | 45206 | 2200 | 10280 | 61894 | 1821841 | 773750 | 63622 | 119 | 423053 |  |
| Developed, Medium Intensity | 21650 | 1724 | 5649 | 77900 | 665745 | 560277 | 108449 | 53 | 265460 |  |
| Developed, Open Space | 74063 | 2207 | 783 | 40578 | 4787863 | 1344846 | 26560 | 417 | 526983 |  |
| Emergent Herbaceous Wetlands | 19103 | 0 | 73 | 1002 | 6568354 | 6633 | 679 | 23 | 3808 |  |
| Evergreen Forest | 98703 | 0 | 69 | 864 | 21907571 | 17068 | 835 | 0 | 2815 |  |
| Hay/Pasture | 185159 | 0 | 1750 | 3625 | 11828949 | 217675 | 12500 | 2821 | 12619 |  |
| Herbaceous | 280114 | 0 | 437 | 1918 | 26798222 | 90181 | 21766 | 5834 | 10182 |  |
| Mixed Forest | 53777 | 0 | 41 | 871 | 10560586 | 13022 | 396 | 15 | 2224 |  |
| Open Water | 24629 | 0 | 161 | 44669 | 12550281 | 3641 | 560 | 18 | 589079 |  |
| Perennial Snow/Ice | 0 | 0 | 0 | 0 | 1746 | 0 | 0 | 0 | 0 |  |
| Shrub/Scrub | 272698 | 0 | 337 | 599 | 44504663 | 127629 | 27515 | 7837 | 7820 |  |
| Unclassified | 5 | 0 | 0 | 0 | 0 | 0 | 0 | 0 | 0 |  |
| Woody Wetlands | 87164 | 0 | 228 | 1258 | 26672574 | 15631 | 564 | 69 | 5869 |  |
| **Cramer’s V** | .21 |  | | | | | | | | |

**Table S3-3. Total percent land cover from 9 NWELD samples according to the Cramer’s V analysis for NLCD**

| **NLCD Classification** | **Decimal percent** |
| --- | --- |
| Shrub/Scrub | 0.19833357 |
| Deciduous Forest | 0.12459625 |
| Herbaceous | 0.12005557 |
| Woody Wetlands | 0.11817899 |
| Evergreen Forest | 0.0971961 |
| Cultivated Crops | 0.09078442 |
| Open Water | 0.05830126 |
| Hay/Pasture | 0.05411857 |
| Mixed Forest | 0.04690796 |
| Developed, Open Space | 0.03002332 |
| Emergent Herbaceous Wetlands | 0.02912043 |
| Developed, Low Intensity | 0.01412836 |
| Barren Land | 0.00753156 |
| Developed, Medium Intensity | 0.00703073 |
| Developed, High Intensity | 0.00368518 |
| Perennial Snow/Ice | 7.7041E-06 |
| Unclassified | 2.2062E-08 |

**Table S3-4. Associations between 9 NWELD samples and the NWALT raster dataset explained by number of NWALT pixels per NWELD layer**

| **NWALT Layers** | **NWELD Layer (Number of Pixels)** |  | | | | | | | | |  |
| --- | --- | --- | --- | --- | --- | --- | --- | --- | --- | --- | --- |
|  | Hydrocarbon Gas Liquid Pipeline | | Hydropower Plant | Natural Gas Storage | Petroleum Powerplant | Small Rivers | Solar Farm | Substations | Uranium Insitu Leaching | Wastewater Treatment Plant |  |
| Anthropogenic Other | 84 | | 0 | 8 | 140 | 10442 | 2835 | 262 | 0 | 1204 |  |
| Commercial/Services | 3413 | | 204 | 403 | 34926 | 148124 | 132315 | 12287 | 0 | 18905 |  |
| Crops | 126666 | | 47 | 889 | 2500 | 5399962 | 405502 | 12248 | 720 | 38863 |  |
| Developed, Other | 2791 | | 78 | 312 | 3448 | 184513 | 85109 | 2955 | 19 | 31719 |  |
| Grazing Potential | 7835 | | 5 | 48 | 75 | 740031 | 795 | 523 | 47 | 2342 |  |
| Industrial/Military | 2732 | | 130 | 1187 | 19850 | 69169 | 175353 | 24348 | 2 | 98383 |  |
| Low Use | 218006 | | 387 | 1609 | 3560 | 34718404 | 60704 | 20712 | 3508 | 61839 |  |
| Major Transportation | 3997 | | 129 | 169 | 31 | 234755 | 463 | 3929 | 0 | 10186 |  |
| Mining/Extraction | 441 | | 4 | 136 | 759 | 31772 | 858 | 1041 | 0 | 2974 |  |
| Pasture/Hay | 45122 | | 60 | 848 | 2252 | 3248909 | 165423 | 6132 | 851 | 35173 |  |
| Recreation | 1223 | | 53 | 41 | 0 | 102963 | 103 | 922 | 0 | 30745 |  |
| Residential, High Density | 3352 | | 228 | 93 | 0 | 116473 | 234 | 3178 | 0 | 5583 |  |
| Residential, Low-Medium Density | 8566 | | 265 | 441 | 4 | 362582 | 475 | 7534 | 0 | 23375 |  |
| Urban Interface High | 2421 | | 40 | 46 | 9 | 285804 | 21928 | 1275 | 0 | 14470 |  |
| Urban Interface Low Medium | 12240 | | 76 | 81 | 7 | 1622269 | 3905 | 4077 | 0 | 8794 |  |
| Very Low Use, Conservation | 1050 | | 6 | 0 | 123 | 1704817 | 6 | 33 | 0 | 981 |  |
| Water | 6109 | | 36 | 47 | 11271 | 2796222 | 105 | 401 | 2 | 127794 |  |
| Wetlands | 9488 | | 2 | 44 | 394 | 2657621 | 118 | 193 | 1 | 4892 |  |
| **Cramer’s V** | .203 | |  | | | | | | | | |

**Table S3-5. Total percent land cover from 9 NWELD samples according to the Cramer’s V analysis for NWALT**

| **NWALT Classification** | **Decimal percent** |
| --- | --- |
| Low Use | 0.61929095 |
| Crops | 0.10567327 |
| Pasture/Hay | 0.06185668 |
| Water | 0.05192396 |
| Wetlands | 0.04717218 |
| Very Low Use, Conservation | 0.03012761 |
| Urban Interface Low Medium | 0.02914689 |
| Grazing Potential | 0.01326698 |
| Residential, Low-Medium Density | 0.00711693 |
| Industrial/Military | 0.00690359 |
| Commercial/Services | 0.00618743 |
| Urban Interface High | 0.00575354 |
| Developed, Other | 0.00548794 |
| Major Transportation | 0.0044769 |
| Recreation | 0.00240119 |
| Residential, High Density | 0.00227925 |
| Mining/Extraction | 0.00067041 |
| Anthropogenic Other | 0.0002643 |

**Table S3-6. Associations between 9 NWELD samples and the NLUD raster dataset explained by number of NLUD pixels per NWELD layer**

| **NLUD Layers** | **NWELD Layers (Number of Pixels)** |  | | | | | | | | |  |
| --- | --- | --- | --- | --- | --- | --- | --- | --- | --- | --- | --- |
|  | Hydrocarbon Gas Liquid Pipeline | | Hydropower Plant | Natural Gas Storage | Petroleum Powerplant | Small Rivers | Solar Farm | Substations | Uranium Insitu Leaching | Wastewater Treatment Plant |  |
| Agricultural conservation | 589 | | 1 | 2 | 0 | 92309 | 1090 | 49 | 0 | 49 |  |
| Airports (developed) | 2083 | | 14 | 325 | 2088 | 35860 | 54019 | 3444 | 0 | 15304 |  |
| Aquacultures | 540 | | 0 | 12 | 1 | 10543 | 8 | 4 | 0 | 432 |  |
| Archaeology, historic site, scenic area | 3717 | | 0 | 0 | 0 | 163555 | 107 | 150 | 0 | 785 |  |
| Areas of Critical Env. Concern, research Natural Area | 2660 | | 0 | 0 | 13 | 837008 | 942 | 822 | 0 | 647 |  |
| Bay inlet | 0 | | 0 | 0 | 51 | 22051 | 80 | 5 | 0 | 831 |  |
| Boat/fishing access | 35 | | 0 | 9 | 0 | 4423 | 28 | 1 | 0 | 53 |  |
| Campground/ranger station | 0 | | 22 | 0 | 276 | 49388 | 1079 | 133 | 0 | 2136 |  |
| Canal/ditch | 576 | | 92 | 0 | 184 | 213382 | 4999 | 94 | 0 | 2433 |  |
| Confined animal feeding | 651 | | 7 | 38 | 1071 | 19705 | 11239 | 1881 | 0 | 2629 |  |
| Conservation area (BLM) | 44780 | | 62 | 75 | 0 | 14533367 | 53859 | 4205 | 776 | 548 |  |
| Corps of Engineers dam | 483 | | 0 | 0 | 0 | 80485 | 12 | 296 | 0 | 4399 |  |
| Cropland/row crops | 456897 | | 101 | 2893 | 4482 | 16523909 | 1147897 | 42583 | 1717 | 93587 |  |
| Dense urban (>0.1 ac) | 856 | | 0 | 26 | 6680 | 31095 | 4075 | 1641 | 0 | 5655 |  |
| Dense urban (>0.1 ac) | 0 | | 148 | 0 | 0 | 0 | 0 | 0 | 0 | 0 |  |
| Desginated recreation area | 0 | | 0 | 0 | 0 | 11243 | 0 | 0 | 0 | 72 |  |
| Entertainment (stadiums,amusement, etc.) | 158 | | 0 | 0 | 42 | 5418 | 372 | 120 | 0 | 1344 |  |
| Estuary & complex channels | 0 | | 0 | 0 | 2183 | 149037 | 876 | 10 | 0 | 10917 |  |
| Exurban (2.5-10 ac) | 75277 | | 805 | 2008 | 23804 | 5944097 | 355697 | 39741 | 0 | 209487 |  |
| Factory, plant | 5078 | | 215 | 393 | 37850 | 159917 | 177212 | 30125 | 0 | 80594 |  |
| Fish & Wildlife Service refuge | 2402 | | 0 | 0 | 30 | 732378 | 3707 | 112 | 0 | 3105 |  |
| General agricultural | 4503 | | 10 | 2 | 1030 | 614552 | 4137 | 537 | 0 | 13822 |  |
| General park | 50354 | | 160 | 415 | 1194 | 19313815 | 35206 | 4942 | 318 | 9802 |  |
| Golf course | 2264 | | 52 | 0 | 368 | 196461 | 5602 | 356 | 0 | 33904 |  |
| Government/public | 228 | | 26 | 62 | 2335 | 12633 | 5528 | 974 | 0 | 4261 |  |
| Grazed | 653215 | | 705 | 5811 | 11634 | 70851001 | 385730 | 60046 | 12162 | 132971 |  |
| Highways, railways | 9822 | | 591 | 674 | 7038 | 706723 | 131807 | 5222 | 8 | 69751 |  |
| Lake | 0 | | 65 | 400 | 9648 | 3150700 | 13755 | 605 | 44 | 145550 |  |
| Marine Protected Area | 257 | | 0 | 0 | 114 | 130921 | 79 | 207 | 0 | 1666 |  |
| Medical (hospitals, nursing home, etc.) | 1109 | | 39 | 49 | 8238 | 40290 | 16682 | 3319 | 0 | 6990 |  |
| Military/DOD (training) | 5270 | | 0 | 68 | 1622 | 516783 | 112251 | 1955 | 0 | 96569 |  |
| Military/DOD/DOE (dev) | 115 | | 24 | 0 | 0 | 1338741 | 88357 | 2005 | 0 | 23835 |  |
| Mining strip mines, quarries, gravel pits | 43 | | 0 | 0 | 977 | 18103 | 1366 | 297 | 0 | 805 |  |
| Municipal watershed | 33 | | 3 | 0 | 0 | 142903 | 2370 | 88 | 0 | 133 |  |
| Natural park | 2819 | | 82 | 5 | 1269 | 959633 | 11567 | 1360 | 0 | 12885 |  |
| Nature reserve | 8542 | | 13 | 0 | 474 | 4222291 | 11921 | 1456 | 0 | 9736 |  |
| Office | 4197 | | 232 | 175 | 21198 | 159258 | 194875 | 17342 | 0 | 41011 |  |
| Open ocean | 2900 | | 8 | 0 | 8512 | 1247662 | 559 | 19 | 0 | 47633 |  |
| Orchards | 213 | | 0 | 12 | 15 | 124592 | 47831 | 333 | 1 | 797 |  |
| Pastureland | 171104 | | 161 | 2906 | 3580 | 12620289 | 434189 | 19037 | 5109 | 68292 |  |
| Playa | 0 | | 12 | 4 | 62 | 582320 | 1590 | 78 | 0 | 5108 |  |
| Prison/penitentiary | 131 | | 0 | 0 | 157 | 3099 | 6510 | 132 | 0 | 336 |  |
| Reservoir | 0 | | 61 | 5 | 3229 | 3840747 | 1111 | 202 | 0 | 99525 |  |
| Resort/ski area | 225 | | 0 | 0 | 0 | 25666 | 1510 | 186 | 0 | 964 |  |
| Retail/Shopping centers | 4402 | | 262 | 308 | 20209 | 196639 | 103919 | 14660 | 0 | 32736 |  |
| River | 5552 | | 222 | 51 | 15109 | 9476755 | 3121 | 431 | 0 | 170498 |  |
| Rural (10-40 ac) | 119895 | | 324 | 2254 | 5717 | 13032771 | 252872 | 22506 | 0 | 77165 |  |
| Rural buildings, cemetery | 5383 | | 378 | 2068 | 30848 | 198475 | 127819 | 44735 | 0 | 111466 |  |
| Schools (dev) | 19 | | 23 | 0 | 1970 | 13735 | 26757 | 945 | 0 | 1408 |  |
| Schools (undeveloped) | 1208 | | 37 | 95 | 10330 | 40489 | 27856 | 2058 | 0 | 6342 |  |
| Sod & switch grass | 76 | | 0 | 3 | 2 | 42499 | 5955 | 105 | 0 | 269 |  |
| Suburban (1-2.5 ac) | 23608 | | 826 | 1167 | 28471 | 1657668 | 153105 | 33313 | 0 | 147284 |  |
| Swamp/Marsh | 0 | | 0 | 0 | 0 | 168863 | 111 | 27 | 0 | 77 |  |
| Timber harvest | 3890 | | 79 | 1928 | 4181 | 468388 | 36197 | 14245 | 277 | 11533 |  |
| Undeveloped | 2507 | | 5 | 0 | 665 | 63841 | 33893 | 537 | 0 | 24225 |  |
| Urban park | 1746 | | 63 | 4 | 850 | 320739 | 4549 | 1017 | 0 | 18801 |  |
| Urban(0.1-1) | 21833 | | 1029 | 1035 | 29598 | 543341 | 74926 | 23145 | 0 | 104966 |  |
| Wash | 335 | | 43 | 14 | 0 | 966602 | 782 | 17 | 0 | 218 |  |
| Wetlands | 83152 | | 0 | 364 | 3303 | 20053429 | 29313 | 2848 | 183 | 38804 |  |
| Wetlands | 0 | | 36 | 0 | 0 | 0 | 0 | 0 | 0 | 0 |  |
| Wild & Scenic river | 70 | | 0 | 4 | 0 | 165651 | 906 | 38 | 0 | 768 |  |
| Wilderness | 643 | | 22 | 0 | 0 | 6167768 | 54 | 61 | 0 | 41 |  |
| Wildlife conservation | 830 | | 4 | 0 | 8 | 814097 | 6017 | 215 | 0 | 737 |  |
| **Cramer’s V** | .174 | |  | | | | | | | | |

**Table S3-7. Total percent land cover from 9 NWELD samples according to the Cramer’s V analysis for NLUD**

| **NLUD Classification** | **Decimal percent** |
| --- | --- |
| Grazed | 0.32247974 |
| Wetlands | 0.09038233 |
| General park | 0.08682636 |
| Cropland/row crops | 0.08171888 |
| Conservation area (BLM) | 0.06545747 |
| Rural (10-40 ac) | 0.06043036 |
| Pastureland | 0.05958591 |
| River | 0.04325056 |
| Exurban (2.5-10 ac) | 0.0297419 |
| Wilderness | 0.027585 |
| Nature reserve | 0.01902519 |
| Reservoir | 0.01764091 |
| Lake | 0.01484997 |
| Suburban (1-2.5 ac) | 0.00914691 |
| Military/DOD/DOE (dev) | 0.00649794 |
| Open ocean | 0.00584602 |
| Natural park | 0.00442543 |
| Wash | 0.0043288 |
| Highways, railways | 0.00416614 |
| Areas of Critical Env. Concern, research Natural Area | 0.00376571 |
| Wildlife conservation | 0.00367545 |
| Urban(0.1-1) | 0.00357691 |
| Fish & Wildlife Service refuge | 0.00331692 |
| Military/DOD (training) | 0.00328465 |
| General agricultural | 0.00285569 |
| Playa | 0.0026347 |
| Timber harvest | 0.00241801 |
| Rural buildings, cemetery | 0.0023306 |
| Factory, plant | 0.0021974 |
| Office | 0.00195996 |
| Retail/Shopping centers | 0.0016686 |
| Urban park | 0.00155517 |
| Golf course | 0.0010688 |
| Canal/ditch | 0.00099168 |
| Orchards | 0.00077718 |
| Swamp/Marsh | 0.00075609 |
| Archaeology, historic site, scenic area | 0.00075267 |
| Wild & Scenic river | 0.00074875 |
| Estuary & complex channels | 0.00072901 |
| Municipal watershed | 0.00065079 |
| Marine Protected Area | 0.00059585 |
| Undeveloped | 0.00056199 |
| Airports (developed) | 0.00050593 |
| Agricultural conservation | 0.00042075 |
| Schools (undeveloped) | 0.00039538 |
| Corps of Engineers dam | 0.00038313 |
| Medical (hospitals, nursing home, etc.) | 0.00034306 |
| Campground/ranger station | 0.00023716 |
| Dense urban (>0.1 ac) | 0.00022372 |
| Sod & switch grass | 0.00021871 |
| Schools (dev) | 0.00020059 |
| Confined animal feeding | 0.00016645 |
| Resort/ski area | 0.00012768 |
| Government/public | 0.00011648 |
| Bay inlet | 0.00010293 |
| Mining strip mines, quarries, gravel pits | 9.6552E-05 |
| Aquacultures | 5.1605E-05 |
| Designated recreation area | 5.0599E-05 |
| Prison/penitentiary | 4.6351E-05 |
| Entertainment (stadiums ,amusement, etc.) | 3.3333E-05 |
| Boat/fishing access | 2.0342E-05 |
| Dense urban (>0.1 ac) | 6.6183E-07 |
| Wetlands | 1.6099E-07 |

**Table S3-8. Description of the accuracy assessment of NWELD by comparison to aerial imagery. Accuracy is measured by the average value of 10 samples per NWELD layer. The average is calculated for each layer/energy type and measured on a scale of 1 to 3. A score of 1 indicates that NWELD poorly represents the energy use, whereas a score of 3 conveys that NWELD represents the energy use exceedingly well.**

| **Energy** | **Layer** | **Average per Layer** | **Standard Deviation per Layer** | **Average per Energy Type** |
| --- | --- | --- | --- | --- |
| Coal | Surface Coal Mines | 1.5 | 0.71 | 1.8 |
|  | Coal Power Plant | 2.1 | 0.88 |  |
| Hydropower | Hydro Dams | 1.6 | 0.84 | 1.77 |
|  | Hydro Plants | 1.4 | 0.84 |  |
|  | Hydro Dam Plants | 2.3 | 0.67 |  |
| Natural Gas/Oil | Oil and Gas Wells | 2 | 0.94 | 2.5 |
|  | Natural Gas/ Petroleum Plant | 3 | 0 |  |
| Natural Gas | Natural Gas Processing Plant | 2.1 | 0.88 | 2.1 |
|  | Natural Gas Storage Facility | 2.2 | 0.79 |  |
|  | Natural Gas Power Plant | 2 | 0.67 |  |
| Oil | Petroleum Refinery | 2.4 | 0.52 | 2.3 |
|  | Petroleum Reserves | 3 | 0 |  |
|  | Petroleum Power Plant | 1.5 | 0.53 |  |
| Nuclear | Uranium In-situ Leaching Plant | 3 | 0 | 2.97 |
|  | Uranium Mills and Heap Leach Facilities | 3 | 0 |  |
|  | Nuclear Power Plant | 2.9 | 0.32 |  |
| Solar | Solar Farms | 1.6 | 0.7 | 1.6 |
| Wind | Wind Farms | 2.8 | 0.63 | 2.8 |
| General Renewable Metal Processing Plants | Zinc metal processing plant | 3 | 0 | 3 |
|  | silver metal processing plant | 3 | 0 |  |
|  | Nickel metal processing plant | 3 | 0 |  |
|  | magnesium metal processing plant | 3 | 0 |  |
|  | lead metal processing plant | 3 | 0 |  |
|  | iron metal processing plant | 3 | 0 |  |
|  | gold metal processing plant | 3 | 0 |  |
|  | copper metal processing plant | 3 | 0 |  |
|  | cobalt metal processing plant | 3 | 0 |  |
| Biomass | Biodiesel Refinery | 1.9 | 0.88 | 2.26 |
|  | Ethanol Refineries | 1.7 | 0.82 |  |
|  | Municipal Landfills with Gas | 3 | 0 |  |
|  | Landfills with Waste and Gas | 2.4 | 0.7 |  |
|  | Municipal Landfills | 2 | 0.82 |  |
|  | Municipal Waste Plant | 3 | 0 |  |
|  | Mills | 1.1 | 0.32 |  |
|  | Wood waste plant | 3 | 0 |  |
| Transmission | Substations | 2.5 | 0.53 | 2.75 |
|  | Transmission Lines | 3 | 0 |  |
| Infrastructure | Railroad | 2.8 | 0.63 | 1.93 |
|  | Flood Control Dams | 1.9 | 0.99 |  |
|  | Irrigation Dams | 2.1 | 0.88 |  |
|  | Navigation Dams | 2.2 | 1.03 |  |
|  | Water Supply Dams | 1.5 | 0.85 |  |
|  | Recreation Dams | 1.6 | 0.84 |  |
|  | Multi-use Dams | 1.4 | 0.7 |  |
| Water Sources | Waterbodies | 2.3 | 0.67 | 2.37 |
|  | Large and Navigable Rivers | 3 | 0 |  |
|  | Small Rivers | 3 | 0 |  |
|  | Hydro Power Reservoirs | 2.7 | 0.48 |  |
|  | Ocean | 2.2 | 0.63 |  |
|  | Wastewater Treatment Plant | 1 | 0 |  |

**Validation 4.** The fourth validation in the primary document takes energy consumption in Quads BTU from the U.S. Energy Information Administration (EIA), calculates the land transformation of the lower 48 states in squared kilometers per terawatt-hours, and compares this value to mathematically calculated values from Fthenakis and Kim 2009 and Jordaan et al 2017.

First, we downloaded the 2020 primary energy consumption by sector in Quads BTU from EIA (<https://www.eia.gov/totalenergy/data/annual/>). Then we converted Quads BTU into Megawatt-hours per the following sectors: coal (2690746454.03), natural gas (9242943110.51), nuclear (2418151380.32), hydroelectric (733471311.89), solar (355041534.1), and wind (868876886.94). We obtained energy consumption information for Alaska(<https://www.eia.gov/state/?sid=AK>)and Hawaii (<https://www.eia.gov/state/?sid=HI>) per sector in BTU’s and converted that data to Megawatt-hours. Hawaii’s consumption in MWh for 2020 is the following: coal (4161609.383), natural gas (0), nuclear (0), hydroelectric (234456.867), solar (5568350.583), wind (5568350.583). Alaska’s consumption in MWh for 2020 is the following: coal (4982208.416), natural gas (103835084.825), nuclear (0), hydroelectric (4249530.708), solar (439606.625), wind (439606.625). Then we subtracted energy consumed in Alaska and Hawaii from the national values to obtain the energy consumed in the lower 48 states.

Then we looked at NWELD layers and cross referenced them to Fthenakis and Kim 2009 as well as Jordaan et al. 2017 to identify exact land uses that could be compared. The following NWELD layers were chosen for coal: (16) surface coal mines, (17) coal fired power plant, and (82) railways. The following NWELD layers were chosen for natural gas: (27) Natural gas power plant, (22) oil and gas wells, (25) natural gas processing plant, (28) natural gas pipes, and (26) natural gas storage. The following NWELD layers were chosen for nuclear: (34) uranium mines, (37) nuclear power plant, (35) insitu leaching plants, and (36) mills and heap leach facilities. The following NWELD layers were chosen for hydroelectric: (18) hydro dams, (19) hydro plants, (20) hydro dam plants, and (21) reservoirs. The only NWELD layers used for wind and solar were (45) wind farms and (43) solar farms. The areas of the rasterized layers were calculated in ArcMap in kilometers squared and summed according to energy sector. The water sources of each power plant and processing plant were included in the summation. However, only 70% of the area of railways were included in the summation, because according to EIA “trains transport 70% of coal deliveries” (<https://www.eia.gov/energyexplained/coal/mining-and-transportation.php>). This resulted in the following total areas: coal (12075.53472 km2), natural gas (15922.8144 km2), nuclear (2456.7453 km2), hydroelectric (25790.3865 km2), wind (56.7km2) and solar (3802.3488 km2).

With the energy consumption for the lower 48 states and the total area per energy sector calculated, we went through a series of calculations to arrive at the desired outcome of squared kilometers per Terawatt-hour. First, we took the total area per sector and applied a percentage representing how much of the land was used to produce a domestically available product. This percentage only applied to coal (87%) and natural gas (82%) and was obtained from EIA. According to EIA, the US exported about 13% of the total coal production, therefore 87% of the total coal production is used domestically (<https://www.eia.gov/energyexplained/coal/imports-and-exports.php>). For natural gas, in 2020 the US exported 5.28 trillion cubic feet(<https://www.eia.gov/energyexplained/natural-gas/imports-and-exports.php>) out of a total of 30.5 trillion cubic feet consumed (<https://www.eia.gov/energyexplained/natural-gas/use-of-natural-gas.php>). Therefore, the export percentage is 5.28/30.5=0.1731 or 17.31% and 82.7% of natural gas is used domestically. For the sectors nuclear, hydroelectric, wind, and solar, it was assumed that 100% of the land is used to produce domestic products.

For coal, nuclear, hydroelectric, solar, and wind we took the total adjusted area and divided by the energy consumed for the lower 48 states to obtain land transformed in km2/MWh, which we then converted to km2/TWh. This conversion resulted in the following: coal (3.92), nuclear (1.02), hydroelectric (35.38), solar (10.98), and wind (0.07). We are assuming that the following sectors are used 100% for electricity production.

However, since natural gas is used for heating as well as electricity production, the sector had to undergo a separate set of calculations. The energy consumed for the lower 48 states in MWh was multiplied by the percent of natural gas used for electricity from EIA (38%) (<https://www.eia.gov/energyexplained/natural-gas/use-of-natural-gas.php>). Then the area of land for natural gas was multiplied by the electricity percentage after being multiplied by the domesticity percentage. Then the adjusted area was divided by the adjusted energy to provide the total land transformed by natural gas in km2/MWh, which was converted to km2/TWh. The result is the following: natural gas (1.43km2/TWh).

With NWELD’s calculations finished, we delved into Fthenakis and Kim 2009 as well as Jordaan et al. 2017 to obtain land transformation calculations with which to compare. For coal we found the following m2/GWh values from Fthenakis and Kim 2009: surface coal mines (400), coal fired power plant (9.1), and railways (55). For natural gas we found the following m2/GWh values from Fthenakis and Kim 2009: Natural gas power plant (5), oil and gas wells (75), natural gas pipes (127), and natural gas storage (61); and the following m2/MWh value from Jordaan et al. 2017: natural gas processing plant (0.0023). For nuclear we found the following m2/GWh values from Fthenakis and Kim 2009: uranium mines (29), nuclear power plant (47), milling (10), conversion (2), enrichment (1), and fabrication (1). For hydroelectric, wind, and solar we averaged the US m2/GWh values found in Fthenakis and Kim 2009 which resulted in 13675, 1950, and 388, respectively.

We converted the values to km2/TWh and summed them according to sector. The result is the following: coal (0.46), natural gas (0.27), nuclear (0.09), hydroelectric (13.68), solar (0.39), and wind (1.95).
