# Supplementary material for: U.S. national water and energy land dataset for integrated multisector dynamics research
Source: Sci Data. 2022 Apr 20;9:183. doi: 10.1038/s41597-022-01290-w (PMC9021314; doi:10.1038/s41597-022-01290-w)
Supplement: Supplementary file 3 — Supplementary Table 1 [file 41597_2022_1290_MOESM3_ESM.docx]

Supplementary Table 1

NWELD data Sources with Corresponding Layers (from Table 1) and Geometry

| **Data Source** | **Description** | **NWELD Corresponding Layers (Codes)** | **Geometry** |
| --- | --- | --- | --- |
| EIA- U.S. Energy Information Administration Layer Information for Interactive Maps^a^ | coal surface mines 2020  coal power plant 2020  HGL pipelines 2019  natural gas power plant 2020  petroleum power plant 2020  crude oil pipelines 2020  petroleum pipelines 2018  uranium in-situ leach plant 2020  uranium mills 2020  nuclear power plant 2020  solar farm 2020  biodiesel refinery 2019  ethanol refinery 2019  municipal landfills with gas  municipal waste plants  wood waste plant | 16  17  22  26  30  31  32  34  35  36  42  70  77  78  81  85 | point  point  line  point  point  line  line  point  point  point  point  point  point  point  point  point |
| HydroSource- Oak Ridge National Laboratory Hydropower Data^b^ | hydro powered dams  hydro powered plants  hydropower reservoirs | 18  19  99 | point  point  polygon |
| BT16 (Oak Ridge National Laboratory Billion Ton 2016)^c^ | Biomass crops per county | 64-69, 71-76 | polygon |
| HIFLD- Homeland Infrastructure Foundation Level Data, Open Source^d^ | oil and natural gas wells  natural gas processing plants  natural gas storage facilities  natural gas pipelines  petroleum refineries  DOE petroleum reserves  substations  transmission lines  railroads  large and navagable rivers  wastewater treatment  non-ferrous mines  ferrous mines  non-ferrous processing plants  ferrous processing plants  solid waste landfill facilities  coal fields | 21  24  25  27  28  29  86  87  88  97  101  40, 46-50  43, 60, 62  51-52, 55-58  53-54, 59  79-80 | point  point  point  line  point  point  point  line  line  polygon  point  point  point  point  point  point  polygon |
| US WTD (U.S. Wind Turbine Database)^e^ | Wind turbines | 44 | point |
| MRDS – Mineral Resources Data System^f^ | Uranium and mineral mines | 33, 37-41, 43, 45-50, 60-63 | point |
| TIGER - U.S. Census 2019-Topologically Integrated Geographic Encoding and Referencing Data^g^ | primary and secondary roads | 89 | line |
| NABD - National Anthropogenic Barrier Dataset^h^ | flood control dams  irrigation dams  navigation dams  water supply dams  recreation dams  multi-use dams | 90  91  92  93  94  95 | point  point  point  point  point  point |
| NHDplus V2 - National Hydrography Dataset Plus^i^ | waterbodies  small network rivers | 96  98 | polygon  line |
| ArcGIS Hub Dataset^j^ | metal mines  coastline ocean boundaries  block groups | 39-41, 61, 63  100  Many | point  polygon  polygon |
| Biofuels Atlas- National Renewable Energy Laboratory Biofuel Atlas Dataset^k^ | Biofuels Atlas Dataset- interactive map that provides geospatial data for the extraction and production of biofuels | 71-76, 82-84 | polygon |
| CDL - United States Department of Agriculture Crop Data Layer^l^ | CDL- crop data layer, 30x30m raster | 64-69, 71-76 | raster |
| EROS – Earth Resources Observation and Science^m^ | Conterminous United States Land Cover Projections – Includes lands used for mining | 33, 37-41, 43, 45-50, 60-63 | raster |
| MRLC- Multiresolution Land Characteristics, NLCD 2016, 30x30m^n^ | National Land Cover Dataset 2016 - Main Raster used as the extraction and reclassification of the created datasets | All layers | raster |
| NWALT 2012, 60x60m (James Falcone, 2015)^o^ | National Wall-to-Wall Anthropogenic Land Use Trends Dataset  Secondary raster used as refining tool to aid in the final steps of extracting and reclassifying NLCD | Many layers | raster |
| NLUD 2014, 30x30m (Theobald, 2014)^p^ | National Land Use Dataset  Secondary raster used as refining tool to aid in the final steps of extracting and reclassifying NLCD | Many layers | raster |
| OSM 2020^q^ | OSM 2020- Open Street Map  Organization used to obtain polygons through R code | Many layers | polygon |
| NARWidth- North American River Width Dataset^r^ | North American River Width Dataset is a primary raster used to obtain large and navigable rivers in the US | 97 | line |

^a^EIA: <https://www.eia.gov/maps/layer_info-m.php>.

^b^ORNL: <https://hydrosource.ornl.gov/datasets>.

^c^BT16: https://bioenergykdf.net/bt16-2-download-tool/state

^d^HIFLD: <https://hifld-geoplatform.opendata.arcgis.com/>.

^e^US WTD: https://eerscmap.usgs.gov/uswtdb/

^f^MRDS: https://mrdata.usgs.gov/mrds/.

^g^TIGER: <https://www.census.gov/geographies/mapping-files/time-series/geo/tiger-geodatabase-file.html>.

^h^NABD: <https://nid.sec.usace.army.mil/ords/f?p=105:1>::::::.

^i^NHDPlus V2: <https://www.epa.gov/waterdata/nhdplus-national-data>.

^j^ArcGIS: https://hub.arcgis.com/search?collection=Dataset .

^k^NREL: <https://www.nrel.gov/gis/biomass.html>, <https://maps.nrel.gov/biomass/?aL=0&bL=clight&cE=0&lR=0&mC=40.17887331434696%2C-91.58203125&zL=4>.

^l^CDL: <https://www.nass.usda.gov/Research_and_Science/Cropland/Release/index.php>.

^m^EROS: <https://www.usgs.gov/core-science-systems/eros/lulc/science/acquiring-land-cover-modeling-data-usgs-eros-center?qt-science_center_objects=0#qt-science_center_objects>

^n^MRLC: <https://www.mrlc.gov/data/nlcd-2016-land-cover-conus>.

^o^Falcone: <https://pubs.er.usgs.gov/publication/ds948>.

^p^Theobald: <http://csp-inc.org/public/NLUD2010_20140326.zip>.

^q^OSM: https://www.openstreetmap.org/#map=6/36.280/-115.225.

^r^NAR:<http://gaia.geosci.unc.edu/NARWidth/#:~:text=This%20is%20the%20distribution%20site,Landsat%20TM%20and%20ETM%2B%20imagery>.
