# Supplementary material for: U.S. national water and energy land dataset for integrated multisector dynamics research
Source: Sci Data. 2022 Apr 20;9:183. doi: 10.1038/s41597-022-01290-w (PMC9021314; doi:10.1038/s41597-022-01290-w)
Supplement: Supplementary file 4 — Supplementary Table 2 [file 41597_2022_1290_MOESM4_ESM.docx]

Supplementary Table 2.

Method approaches used to convert the original data into rasterized NWELD Classes. Original datasets follow acronyms in Table 2. MRLC NLCD was used to produce all layers, therefore it is not mentioned in the table. Otherwise specific datasets are noted.

| **NWELD Classes** | **Life Cycle Sub-Layers** | **Description of Data Use** | **Original Data** | **General Method** |
| --- | --- | --- | --- | --- |
| Coal | Surface Coal Mines | Point locations of surface coal mines; rasters providing mining land classes | EIA, EROS, NWALT | Theissen Polygons |
|  | Coal Fired Power Plants | Point locations of coal fired power plants; raster excluding transportation, recreation, residential, and urban interface land classes | EIA, NWALT | OSM/Regression Buffer |
| Hydropower | Hydro Dams | Point locations of hydropower dams | ORNL | OSM/Regression Buffer |
|  | Hydropower Plants | Point locations of hydropower plants | ORNL | OSM/Regression Buffer |
|  | Hydro Dam/Power Plants | A point layer created from identifying the hydropower plants and dams at the same locations | ORNL | OSM/Regression Buffer |
|  | Hydropower Reservoirs | Point layer of hydropower plants and dams; Polygon areas representing hydropower reservoirs | ORNL, NHDPlus V2 | Original Data |
| Natural Gas/Oil | Oil and Gas Wells | Point locations of oil and gas wells | HIFLD | Original Data |
|  | Hydrocarbon Gas Liquid Pipelines | Line networks representing hydrocarbon gas liquid pipelines | EIA | Original Data |
|  | Natural Gas and Petroleum Plant | A point layer created from identifying the natural gas power plants and petroleum power plants found at the same locations | EIA | Manually Digitized Polygons |
| Natural Gas | Natural Gas Processing Plant | Point locations of natural gas processing plants; raster excluding transportation, recreation, residential, and urban interface land classes | HIFLD, NWALT | Manually Digitized Polygons |
|  | Natural Gas Power Plant | Point locations of natural gas power plants | EIA | OSM/Regression Buffer |
|  | Natural Gas Storage Facilities | Point locations of natural gas storage facilities | HIFLD | Manually Digitized Polygons |
|  | Natural Gas Pipelines | Line networks representing natural gas pipelines | HIFLD | Original Data |
| Oil | Petroleum Refinery | Point locations of petroleum refineries; raster providing transportation, commercial, residential, and urban interface land classes | HIFLD, NWALT | Manually Digitized Polygons |
|  | Petroleum Power Plant | Point locations of petroleum power plants; raster excluding transportation, recreation, residential, and urban interface land classes | EIA, NWALT | OSM/Regression Buffer |
|  | Petroleum Reserves | Point locations of petroleum reserves | HIFLD | Manually Digitized Polygons |
|  | Crude Oil Pipelines | Line networks representing crude oil pipelines | EIA | Original Data |
|  | Petroleum Pipelines | Line networks representing petroleum pipelines | EIA | Original Data |
| Nuclear | Uranium Mines | Point locations of uranium mines | USGS, EROS, NWALT | Theissen Polygons |
|  | Uranium Insitu Leaching Plant | Point locations of uranium insitu leaching plants | EIA | Manually Digitized Polygons |
|  | Uranium Mills and Heap Leach Facilities | Point locations of uranium mills and heap leach facilities | EIA | Manually Digitized Polygons |
|  | Nuclear Power Plant | Point locations of nuclear power plants; raster providing industrial and mining land classes | EIA, NWALT | OSM/Regression Buffer |
| Solar | Quartz Mine for Solar Panels | Point locations of quartz mines; rasters providing mining land classes | MRDS, EROS, NWALT | Theissen Polygons |
|  | Cadmium Mine for Solar Panels | Point locations of cadmium mines; rasters providing mining land classes | MRDS, EROS, NWALT | Theissen Polygons |
|  | Gallium Mine for Solar Panels | Point locations of Gallium mines; rasters providing mining land classes | MRDS, ArcGIS, EROS, NWALT | Theissen Polygons |
|  | Germanium Mine for Solar Panels | Point locations of germanium mines; rasters providing mining land classes | MRDS, ArcGIS, HIFLD, EROS, NWALT | Theissen Polygons |
|  | Tellurium Mine for Solar Panels | Point locations of tellurium mines; rasters providing mining land classes | MRDS, ArcGIS, EROS, NWALT | Theissen Polygons |
|  | Solar Farms | Point locations of solar farms; raster providing commercial, industrial, developed, crop, and pasture land classes | EIA, NWALT | OSM/Regression Buffer |
| Wind | Iron Mine for Windmills | Point locations of iron mines; rasters providing mining land classes | MRDS, HIFLD, EROS, NWALT | Theissen Polygons |
|  | Wind Farms | Point locations of wind turbines | US WTD | Original Data |
| General Renewable Mines | Aluminum Metal Mine | Point locations of aluminum mines; rasters providing mining land classes | MRDS, EROS, NWALT | Theissen Polygons |
|  | Copper Metal Mine | Point locations of copper mines; rasters providing mining land classes | MRDS, HIFLD, EROS, NWALT | Theissen Polygons |
|  | Gold Metal Mine | Point locations of gold mines; rasters providing mining land classes | MRDS, HIFLD, EROS, NWALT | Theissen Polygons |
|  | Silver Metal Mine | Point locations of silver mines; rasters providing mining land classes | MRDS, HIFLD, EROS, NWALT | Theissen Polygons |
|  | Zinc Metal Mine | Point locations of zinc mines; rasters providing mining land classes | MRDS, HIFLD, EROS, NWALT | Theissen Polygons |
|  | Lead Metal Mine | Point locations of lead mines; rasters providing mining land classes | MRDS, HIFLD, EROS, NWALT | Theissen Polygons |
| General Renewable Metal Processing Plants | Zinc Metal Processing Plant | Point locations of zinc metal processing plants | HIFLD | Manually Digitized Polygons |
|  | Silver Metal Processing Plant | Point locations of silver metal processing plants | HIFLD | Manually Digitized Polygons |
|  | Nickel Metal Processing Plant | Point locations of nickel metal processing plants | HIFLD | Manually Digitized Polygons |
|  | Magnesium Metal Processing Plant | Point locations of magnesium metal processing plants | HIFLD | Manually Digitized Polygons |
|  | Lead Metal Processing Plant | Point locations of lead metal processing plants | HIFLD | Manually Digitized Polygons |
|  | Iron Metal Processing Plant | Point locations of iron metal processing plants | HIFLD | Manually Digitized Polygons |
|  | Gold Metal Processing Plant | Point locations of gold metal processing plants | HIFLD | Manually Digitized Polygons |
|  | Copper Metal Processing Plant | Point locations of copper metal processing plants | HIFLD | Manually Digitized Polygons |
|  | Cobalt Metal Processing Plant | Point locations of cobalt metal processing plants | HIFLD | Manually Digitized Polygons |
| Mines for Lithium-Ion Batteries (Renewables) | Cobalt Mine | Point locations of cobalt mines; rasters providing mining land classes | MRDS, HIFLD, EROS, NWALT | Theissen Polygons |
|  | Lithium Mine | Point locations of lithium mines; rasters providing mining land classes | MRDS, ArcGIS, EROS, NWALT | Theissen Polygons |
|  | Nickel Mine | Point locations of nickel mines; rasters providing mining land classes | MRDS, HIFLD, EROS, NWALT | Theissen Polygons |
|  | Manganese Mine | Point locations of manganese mines; rasters providing mining land classes | MRDS, ArcGIS, EROS, NWALT | Theissen Polygons |
| Biomass | Soybean  Rapeseed  Canola  Mustard  Camelina  Safflower  Sunflower | Polygon areas of crop for Biodiesel Refineries; raster providing crop designation | BT16, CDL | Original Data |
|  | Biodiesel Refinery | Point locations of biodiesel refineries; raster providing transportation, commercial, residential, and urban interface land classes | EIA, NWALT | Manually Digitized Polygons |
|  | Corn  Barley  Rice Straw  Sorghum  Sugarcane Bagasse  Switchgrass | Polygon locations of crop for Ethanol Refineries; raster providing crop designation | NREL, CDL | Original Data |
|  | Ethanol Refinery | Point locations of ethanol refineries; raster providing transportation, commercial, residential, and urban interface land classes | EIA, NWALT | Manually Digitized Polygons |
|  | Municipal Landfills with Gas | Point locations of municipal landfills with gas | EIA | Manually Digitized Polygons |
|  | Landfills with Waste and Gas | Point locations of landfills with waste and gas; raster providing urban interface, crop, industrial, mining, pasture, and low use land classes | EIA, HIFLD, NWALT, NLUD | OSM/Regression Buffer |
|  | Municipal Landfills | Point locations of municipal landfills with waste only; raster providing mining land class; raster providing timber harvest and office land classes | HIFLD, NWALT, NLUD | OSM/Regression Buffer |
|  | Municipal Waste Plant | Point locations of waste-to-energy plants | EIA | Manually Digitized Polygons |
|  | Bio-prime Mills  Bio-Forests  Both Mills and Forests | Point locations of woody solids from bio-prime mills and bio-forests; raster providing mechanically disturbed lands | NREL, EROS | Original Data |
|  | Wood Waste Plant | Point locations of wood waste plants | EIA | Manually Digitized Polygons |
| Transmission | Substations | Point locations of substations | HIFLD | OSM/Regression Buffer |
|  | Transmission Lines | Line networks representing transmission lines | HIFLD | Original Data |
| Infrastructure | Railroads | Line networks representing railroad tracks | HIFLD | Original Data |
|  | Primary and Secondary Roads | Line networks representing primary and secondary roads | TIGER | Original Data |
|  | Flood Control Dams | Point locations of flood control dams | NABD | OSM/Regression Buffer |
|  | Irrigation Dams | Point locations of irrigation dams | NABD | OSM/Regression Buffer |
|  | Navigation Dams | Point locations of navigation dams | NABD | OSM/Regression Buffer |
|  | Water Supply Dams | Point locations of water supply dams | NABD | OSM/Regression Buffer |
|  | Recreation Dams | Point locations of recreation dams | NABD | OSM/Regression Buffer |
|  | Multi-Use Dams | Point locations of multi-use dams | NABD | OSM/Regression Buffer |
| Water Sources | Waterbodies | Polygon areas representing waterbodies such as lakes and ponds | NHDPlus V2 | Original Data |
|  | Large and Navigable Rivers | Line networks representing large and navigable rivers in the US such as the Mississippi | NARWidth | Original Data |
|  | Small Network Rivers | Line networks representing small rivers | NHDPlus V2 | Original Data |
|  | Coast Line Ocean | Polygon areas representing the coastal ocean surrounding the US | ArcGIS | Original Data |
|  | Wastewater Treatment Plants | Point locations of wastewater treatment plants; raster providing water, industrial, and developed land classes | HIFLD, NWALT | OSM/Regression Buffer |
