# Supplementary material for: U.S. national water and energy land dataset for integrated multisector dynamics research
Source: Sci Data. 2022 Apr 20;9:183. doi: 10.1038/s41597-022-01290-w (PMC9021314; doi:10.1038/s41597-022-01290-w)
Supplement: Supplementary file 5 — Supplementary File 1 [file 41597_2022_1290_MOESM5_ESM.docx]

Supplementary File 1. Utilized Data and Methodology Examples for NWELD

**U.S. national water and energy land dataset for integrated multisector dynamics research**

Jillian Sturtevant^1^, Ryan A. McManamay^1*^, Christopher R. DeRolph^2^

^1^*Department of Environmental Science, Baylor University, Waco, TX 76798*

^2^Environmental Sciences Division, Oak Ridge National Laboratory, Oak Ridge, TN 37831

*Corresponding Author

[Ryan_McManamay@baylor.edu](mailto:Ryan_McManamay@baylor.edu)

One Bear Place #97622

Waco, Texas 76798-7266

**Methodologies**

1. **Original Data**

**(22) Hydrocarbon Gas Liquid Pipelines,**

**(27) Natural Gas Pipelines,**

**(31) Crude Oil Pipelines,**

**(32) Petroleum Pipelines,**

**(88) Railroad Tracks,**

**(98) Small Network Rivers,**

**(21) Oil and Gas Wells,**

**(44) Wind Turbines**

The original line and point data is projected into USA Contiguous Albers Equal Area Conic USGS and the tool ‘Extract by Mask’ is used to extract NLCD raster pixels from under the line or polygon. Every ‘Extract by Mask’ is snapped to NLCD to assure the resulting pixel size will match that of NLCD, 30x30m. The extracted pixels are reclassified so that every unique classifier of NLCD (11-95) will have the same pixel value; thereby creating raster layers of specified land use with 30-meter width. Hydrocarbon Gas Liquid Pipelines, Natural Gas Pipelines, Crude Oil Pipelines, Petroleum Pipelines, Railroads, and Small Network Rivers are raster lines with a 30-meter width, while Oil/Gas Wells and Wind Turbines are raster points with a 30x30m width and height.

**(97) Large and Navigable Rivers,**

**(100) Ocean**

The shapefile data for the ocean layer is a polygon, while the data for large and navigable rivers is a polyline. For the ocean layer, NLCD pixels are extracted according to the polygon, and the unique classifiers of NLCD (11-95) are reclassified so that 11-Open Water, 90-Woody Wetland, and 95-Emergent Herbaceous Wetlands are given the same value while other classifiers are designated as having no data. For the large and navigable river layer, the polyline data has width associated with each line segment. Therefore, each width was used as an area in the equation ($A=\pi r^{2}$) and the radius was calculated for the width of a buffer. Then the buffer was used to extract NLCD pixels, and the unique classifiers of NLCD (11-95) are reclassified so that 11-Open Water, 90-Woody Wetland, and 95-Emergent Herbaceous Wetlands are given the same value while other classifiers are designated as having no data. The result of these two approaches are raster polygons whose shape are defined by the original datasets (NHD and NAR) and refined with the classifications of NLCD.

**(87) Transmission Lines**

For transmission lines, the line data is separated according to voltage class and periodically sampled according to each class; of which there are 8. For each sampled line the aerial imagery base layer offered from ArcMap is used to record the width of tree cover or bushes cleared for the transmission lines, in meters. For every voltage class the average of the recorded widths is calculated and divided it in half to create a buffer radius. Using each voltage class buffer, the buffered lines are projected into USA Contiguous Albers Equal Area Conic USGS and used to extract NLCD raster pixels. The extracted pixels are reclassified so that every unique classifier of NLCD (11-95) will have the same pixel value; thereby creating raster lines with varying widths in meters.

**(89) Primary and Secondary Roads**

For roads, the line data is separated according to road type and periodically sampled according to each type; of which there are 7. For each sampled line the aerial imagery base layer offered from ArcMap is used to record the width of the roads, in meters. For only 5 of the road types the average of the recorded widths is calculated and divided it in half to create a buffer radius. The other 2 road types are unable to create buffers and are represented by taking the original line data, extracting pixels from NLCD, and reclassifying every unique classifier of NLCD (11-95) as having the same pixel value. Using the 5 road type buffers, the buffered lines are projected into USA Contiguous Albers Equal Area Conic USGS and used to extract NLCD raster pixels. The unique classifiers of NLCD (11-95) are reclassified so that 21-Developed, Open Space, 22-Developed, Low Intensity, 23-Developed, Medium Intensity, and 24-Developed, High Intensity are given the same value while other classifiers are designated as having no data. The two reclassified raster lines are merged to create the final layer, primary and secondary roads with varying widths, in meters.

**(96) Waterbodies**

This non-reservoir water body polygon layer is divided into ‘small’ waterbodies and ‘large’ waterbodies by a 1km^2^(1,000,000m^2^) cut off from the following literature: Downing et al., 2006. Any water body equal to or less than 1,000,000m^2^ is considered a small water body, while any water body greater than 1,000,000m^2^is considered a large water body. A 60m buffer is created for the ‘large’ waterbodies. The ‘small’ waterbodies and buffer for ‘large’ waterbodies are projected into USA Contiguous Albers Equal Area Conic USGS and the tool ‘Extract by Mask’ is used to extract NLCD raster pixels from under the respective polygon designations. For the ‘small’ waterbodies, the extracted pixels are reclassified so that every unique classifier of NLCD (11-95) will have the same pixel value. Whereas for ‘large’ waterbodies, the extracted pixels are reclassified so 11-Open Water is given one value while 90-Woody Wetland and 95-Emergent Herbaceous Wetlands are given the same value, and all other classifiers are designated as having no data. As a result, ‘small’ waterbodies maintain NHD shape and are considered open water. As opposed to buffered ‘large’ waterbodies that use NHD shape as an outline, but have designated values for open water and wetlands as dictated by NLCD.

**(99) Hydropower Reservoirs**

For Hydropower reservoirs the original polygon data is used with no modification. The reservoir polygons are projected into USA Contiguous Albers Equal Area Conic USGS and the tool ‘Extract by Mask’ is used to extract NLCD raster pixels from under the polygons. The extracted pixels are reclassified so 11-Open Water, 90-Woody Wetland, and 95-Emergent Herbaceous Wetlands are given the same value while all other classifiers are designated as having no data. The result is hydropower reservoirs that maintain NHD shape but have no distinction between open water and wetlands.

**(64-69) Biodiesel Crops**

 For soybeans the BT16 county polygons are projected into USA Contiguous Albers Equal Area Conic USGS and the tool ‘Extract by Mask’ is used to extract CDL raster pixels from under the polygons. For the remaining biodiesel crops, biodiesel refineries point data was used to select United States counties; and those counties were used to extract CDL raster pixels. The extracted pixels are reclassified so only the designated crop is selected. Then the tool ‘Extract by Mask’ is used to extract NLCD raster pixels from under the designated raster crop. The result is USDA crops used for biodiesel production that are refined by associated county data.

**(71-76) Ethanol Crops**

NREL county polygons are projected into USA Contiguous Albers Equal Area Conic USGS and the tool ‘Extract by Mask’ is used to extract CDL raster pixels from under the polygons. The extracted pixels are reclassified so only the designated crop is selected. Then the tool ‘Extract by Mask’ is used to extract NLCD raster pixels from under designated raster crop. The result is crops used for ethanol production that are refined by associated county data.

**(82-84) Woody Solids**

NREL county polygons are projected into USA Contiguous Albers Equal Area Conic USGS and broken into just forests, just mills, or both forests and mills. Then the designated polygons are used with the tool ‘Extract by Mask’ to extract NLCD and EROS raster pixels. The NLCD extracted pixels are reclassified so 41- Deciduous Forest, 42- Evergreen Forest, 43- Mixed Forest, 52- Shrub/Scrub are given the same value while all other classifiers are designated as having no data. The EROS (B1_2010, and Historic_2005) extracted pixels are reclassified so 3- Mechanically Disturbed National Forests, 4-Mechanically Disturbed Other Public Lands, and 5-Mechanically Disturbed Private are given the same value while all other classifiers are designated as having no data. Then the tool ‘Extract by Mask’ is used to extract NLCD raster pixels from under the EROS reclassified raster and reclassified so that every unique classifier will have the same pixel value. The two rasters are mosaicked based on their designation and the result is mills, forests, and a combination of mills and forests used for biomass that are refined by associated county data.

1. **OSM/Regression Buffer**

**General Method**

The base of this methodology rests in finding the Open Street Map polygons that represent the area of land use points. Once the polygons are designated, then the geometry of those OSM polygons are calculated in meter squared; the area of the polygons and characteristics of the point data are used to create a regression equation. If there is no x axis value that corresponds to the area to build a regression equation, then the mean of the OSM polygons is used as the area and the radius of the buffer was calculated using ($A=\pi r^{2}$). If the regression equation can be used, then it is used to create a buffer for the points without polygons. The buffer is modified to an appropriate size to fully encompass the desired land use. Then the tool ‘Extract by Mask’ is used to extract pixels from underneath the buffer and reclassified according an appropriate schema. As a mirror of the final buffer step, the OSM polygons that do represent the point data are used to ‘Extract by Mask’ with NLCD, and reclassified so that every unique classifier will have the same pixel value. Finally the two raster layers, buffer and OSM, are mosaicked to create the final land use raster layer.

**(18-20) Hydropower Dams and Plants**

As point shapefiles, hydro dams and hydro plants depend upon one another and therefore are processed together. OSM polygons used for these layers are a merged Shapefile of OSM dam polygons, OSM dam lines buffered with 30m width, and OSM hydro power plant polygons. From this merged layer, the polygons representing the respective areas of hydro dam points and hydro plant points are designated as separate OSM polygon layers. The only way to do this is to first find polygons representing hydro plants and hydro dams found at the same location, and reverse the selection according to the point layer in question. The hydro dams and plants found at the same locations also have a designated OSM polygon layer. However, the calculated geometry of only the separated dam and plant polygon layers, as well as chosen characteristics from the point data, are used to create two separate regression equations; an equation for only hydro dams, and an equation for only hydro plants. There is no equation for the dams and plants found at the same location. From these equations, and proper modifiers to the equation, buffers are created for the dam and plant points that do not have OSM polygons to represent them. At this point the dam buffers and plant buffers that are found at the same location are identified and designated as their own buffer layer, thereby creating three buffer layers; dams only, plants only, and dams and plants found at the same location. These buffer layers are used to extract raster pixels from NLCD, and reclassified so 21-Developed, Open Space, 22-Developed, Low Intensity, 23-Developed, Medium Intensity, and 24-Developed, High Intensity, and 31- Barren Land are given the same value while all other classifiers are designated as having no data. Then the OSM polygons identified as being only hydro dams, only hydro plants, and dams/plants in the same location are used to extract raster pixels from NLCD and reclassified so every extracted pixel has the same value. The resulting rasterized polygons and rasterized buffers are mosaicked according to their designation of either being only hydro dams, only hydro plants, or hydro dams and plants found in the same location.

**(90-95) Flood Control Dam,**

**Irrigation Dam,**

**Navigation Dam,**

**Water Supply Dam,**

**Recreation Dam,**

**Multi-Use Dams**

As point shapefiles, flood control dams, irrigations dams, navigation dams, water supply dams, recreation dams, and multi-use dams depend on one another and are therefore processed together. OSM polygons used for these layers are a merged shapefile of OSM dam polygons and OSM dam lines buffered with 30m widths.  Point data of the six non-hydro dams are used to designate the merged Shapefile of OSM polygons into the respective six layers, points without polygons also being identified. In order to create a buffer for points without polygons, all six designated OSM polygon layers have their areas calculated, and along with characteristics from each layers point data, are used to create six regression equations; one per dam type. These regression equations are modified according to how best model the size of each dam type. Once the best regression equation is identified, the buffers are used to extract raster pixels from NLCD and reclassified so 21-Developed, Open Space, 22-Developed, Low Intensity, 23-Developed, Medium Intensity, and 24-Developed, High Intensity, and 31- Barren Land are given the same value while all other classifiers are designated as having no data. Then, each designated OSM polygon is used to extract raster pixels from NLCD and reclassified so every pixel has the same value. As the final step, the resulting rasterized polygons and rasterized buffers are mosaicked according to their designation; flood control dams, irrigations dams, navigation dams, water supply dams, recreation dams, and multi-use dams.

**(36) Nuclear Power Plant**

The nuclear power plant buffer schema extracts pixels from NWALT and reclassifies them so 11-Open Water, 23- Industrial/Military, and 41-Mines given the same value while all other classifiers are designated as having no data. That rasterized reclassification is used to extract pixels from NLCD and reclassified so 21-Developed, Open Space, 22-Developed, Low Intensity, 23-Developed, Medium Intensity, and 24-Developed, High Intensity, and 31- Barren Land are given the same value while all other classifiers are designated as having no data.

**(86) Substations**

The substation buffer schema extracts pixels from NLCD and reclassifies them so 21-Developed, Open Space, 22-Developed, Low Intensity, 23-Developed, Medium Intensity, 24-Developed, High Intensity, 31-Barren Land, 52-Shrub/Scrub, 71-Grassland/Herbaceous, and 82-Cultivated Crops are given the same value while all other classifiers are designated as having no data.

**(42) Solar Farms**

Solar farms vary not only in the extraction and reclassification schema, but also in the presence of buffers from a regression equation. Instead of finding the regression equation and creating buffers from that equation for points without polygons, census blocks are used as “buffers”. Census blocks are dependent upon population, and it follows the pattern of having smaller polygons in urban areas while having larger polygons in rural areas, which is a similar pattern observed for solar farms. The blocks corresponding to points without polygons are used to extract raster pixels from NWALT and reclassified so that 22-Commercial/Services, 23- Industrial/Military, 27-Developed/Other, 31-Urban Interface High, 43-Crops, and 44-Pasture/Hay are given the same value while all other classifiers are designated as having no data. The exclusion of a large amount of raster classifiers allows the small polygons of the urban areas to exclude residential areas and roads, while the large polygons are able to exclude residential areas, roads, and vast amounts of empty space. This newly reclassified raster from NWALT is used to extract pixels from NLCD, and the extract pixels are reclassified so 21-Developed, Open Space, 22-Developed, Low Intensity, 23-Developed, Medium Intensity, and 24-Developed, High Intensity, are given the same value while all other classifiers are designated as having no data. The OSM polygons of solar farms designated by the solar farm point data are used to extract raster pixels from NLCD. The extracted pixels are reclassified so every unique classifier is given the same value. As a final step, the OSM and buffer rasters of solar farms are mosaicked together.

**(101) Wastewater Treatment Plant**

The wastewater treatment schema uses the mean of the OSM polygons to determine the radius of the buffer. It also utilizes a buffer that is based on the mean plus the standard deviation. This mixed buffer schema extracts pixels from NWALT and reclassified so that 11-Water, 23- Industrial/Military, 27-Developed/Other, and 33-Anthropogenic Other are given the same value while all other classifiers are designated as having no data. Then, the OSM polygons and reclassified buffers are used to extract raster pixels from NLCD and reclassified so every pixel has the same value; except water, which is kept as a separate value. As the final step, the resulting rasterized polygons and rasterized buffers are mosaicked. The final layer represents wastewater treatment plants and their accompanying water sources.

**(17) Coal Fired Power Plants,**

**(24) Natural Gas Processing Plant,**

**(30) Petroleum Power Plant**

This method uses both OSM polygons obtained through R code and drawn polygons. This is because the original point data of the three layers- coal, natural gas, and petroleum power plants- can be found very close to one another and in some cases they are overlapping. Any point that is found within 500 meters of one another is drawn. The drawn and OSM polygons provide the area for the regression equations for each plant. This combined schema extracts pixels from NWALT and reclassified so that 21- Major Transportation, 24- Recreation, 25- Residential/High Density, 26- Residential/Low-Medium Density, 31-Urban Interface High, and 32-Urban Interface Low Medium are designated as having no data while all other classifiers are given the same value. Then, the OSM polygons are reclassified so every pixel has the same value while the buffers are used to extract raster pixels from NLCD and reclassified so 21-Developed, Open Space, 22-Developed, Low Intensity, 23-Developed, Medium Intensity, and 24-Developed, High Intensity, 31- Barren are given the same value, and 11-Water is given its own value. As the final step, the resulting rasterized polygons and rasterized buffers are mosaicked according to the appropriate designation; coal fired power plant, natural gas processing plant, petroleum power plant.

**(79) Landfills with Waste and Gas**

Landfills with waste and gas is developed from point shapefiles of landfills with only waste and landfills with only gas. This is because the original point data of the two layers can be found very close to one another and in some cases are overlapping. Any point that is found within 1000 meters of one another is drawn (7 polygons). The drawn polygons are merged with OSM polygons that have been designated as being both solid waste and gas. The points without polygons used a duel buffer methodology. Then the ‘Extract by Mask’ tool is used to extract NWALT raster pixels from underneath the duel buffer and reclassified so 32- Urban Interface High, 43- Crops, 23- Industrial/Military, 41-Mining/Extraction, 44-Pasture/Hay, and 50-Low Use are given the same value while all other classifiers are designated as having no data. The newly reclassified raster is used to ‘Extract by Mask’ from NLUD and reclassified so 121- Reservoir, 213-Suburban, 214-Exurban, 215-Rural, 222-Retail/Shopping Centers, 231-Factory/Plant, 261-Rural Buildings/Cemetery, 311-Cropland/Row Crops, 312-Pastureland, 321-Grazed, and 341-Timber Harvest are given the same value while all other classifiers are designated as having no data. Then that raster is used to ‘Extract by Mask’ from NLCD and reclassified so 21-Developed, Open Space, 22-Developed, Low Intensity, 23-Developed, Medium Intensity, and 24-Developed, High Intensity, 31- Barren, 52- Shrub/scrub, and 71- Herbaceous are given the same value while all other classifiers are designated as having no data. Then, the OSM polygons are used to extract raster pixels from NLCD and reclassified so every pixel has the same value. As the final step, the resulting rasterized polygons and rasterized buffers are mosaicked. The final layer represents landfills with both solid waste and gas production.

**(80) Municipal Landfills**

The points without polygons for municipal landfills use a triple buffer methodology with a radius calculated from taking the average of the OSM polygons and using the equation ( $A=\pi r^{2}$). The larger buffer is used to ‘Extract by Mask’ from NLCD, NWALT, and NLUD and the rasters are reclassified as the following: NLCD 31- Barren Land, NWALT 41-Mining/Extraction, NLUD 341-Timber Harvest, and NLUD 221-Office. The smaller buffer is used to ‘Extract by Mask’ from NWALT and reclassified as 23-Industrial/Military and 44-Pasture/Hay. The third buffer, from the original regression equation, is used to ‘Extract by Mask’ from NLUD and reclassified as 231-Factory/Plant. The NLUD and NWALT rasters are ‘Extract by Mask’ from NLCD and reclassified so every pixel has the same value. Then, the OSM polygons are used to extract raster pixels from NLCD and reclassified so every pixel has the same value. Finally, rasterized OSM polygons and all rasterized buffers (large, small, and original) are mosaicked. The final layer represents municipal landfills.

1. **Theissen Polygons**

**(16) Surface Coal Mines,**

**(33) Uranium Mines,**

**(37-41, 43, 45-50, 60-63) Various Mines**

The theissen polygons are built from an amalgamation of mine point data. These polygons are separated based on their association with the point data into designated theissen polygons. The coal mine theissen polygons are clipped by coal field polygons since the coal fields and coal theissen polygons were found in approximately the same areas. Using the clipped coal theissen polygons and the other designated theissen polygons the ‘Extract by Mask’ tool is used to extract NWALT and EROS raster pixels. The pixels are reclassified as the following: NWALT, 41-Mines and EROS, 6-Mines. The two raster layers are used to ‘Extract by Mask’ from NLCD then mosaicked together according to the designated mine type.

1. **Manually Digitized Polygons**

**General Method**

There was an attempt to obtain OSM polygons for these layers but the resulting data was minimal and did not suffice. Instead, every point is drawn. Or if there are too many to draw, the original point data that fit certain requirements is periodically sampled for approximately 30 points. The requirement for the point data is the following: it must include a positive value for the category predetermined as the x axis. This requirement is so a regression equation can be calculated in later steps. Once the point data has removed negative values from the category predetermined as the x axis, then the data is exported to an excel file and sampled periodically to obtain approximately 30 points according to FID number. These 30 points are located in the original point data shapefile and separated into their own shapefile. From this sample shapefile, the land use area of the samples is drawn in ArcMap using the aerial imagery and OSM base maps. Again, the drawn polygons focus on developed areas representing the points. If there was confusion as to the location of a point then the name or address of the point data would be put into Google Maps as a verification method. The polygons drawn from the 30-point sample stands in place for the OSM polygons. The area is calculated for the drawn polygons. The area of the polygons is used as the y axis in the buffer regression equation, while the x axis comes from the original point data. The regression equation is calculated using excel, used to create a buffer for the points not included in the sample, and modified via observation to find a buffer size that fits the land use area of a majority of point data. Then, as mentioned before, the buffers are used to extract raster pixels from NWALT or NLCD in an extraction and reclassification schema specific to each layer’s needs. Finally, the ‘buffer’ raster and the ‘drawn’ raster are mosaicked.

**(29) Petroleum Reserves,**

**(34) Uranium In-situ Leaching Plant,**

**(35) Uranium Mills and Heap Leach Facilities**

There is no OSM code for uranium in-situ leaching plants or uranium mills. There was OSM code for petroleum reserves but the data result was not sufficient. The original point data for these layers is minimal; petroleum reserves has 11 points, uranium in-situ leaching plant has 16 points (only 9 points were usable), and uranium mills and heap leach facilities have 4 points. Since the point count of the original data is so small, the points are put into ArcMap and their land use areas are manually digitized using the aerial imagery and OSM base maps. The manually digitized polygons focused on developed areas and attempted to only capture the developed areas of the point, not sparsely used land that may surround the facility. These drawn polygons were used to extract raster pixels from NLCD and the extract pixels are reclassified so every unique classifier has the same value.

**(70) Biodiesel Refinery,**

**(77) Ethanol Refinery,**

**(28) Petroleum Refinery**

Every layer followed this methodology. One difference is that the layers biodiesel plants, ethanol plants, and petroleum refineries are processed together. Any point within 400 meters of one another have their polygon drawn in ArcMap. This allows for buffers of different layers to not overlap. In terms of extraction, the buffers for biodiesel plants, ethanol plants, and petroleum refineries were used to extract raster pixels from NWALT. These pixels are reclassified so 21-Major Transportation, 22-Commercial/Services, 25-Residential, High Density, 26- Residential, Low Medium Density and 32-Urban Interface Low Medium were excluded. The new raster from this exclusion is used to extract raster pixels from NLCD. The extracted pixels are reclassified so 21-Developed, Open Space, 22-Developed, Low Intensity, 23-Developed, Medium Intensity, 24-Developed, High Intensity, and 31-Barren Land are the same value, while all other classifiers are designated as having no data.

**(24) Natural Gas Processing Plants**

The extraction and reclassification schema of the two singularly processed layers are simple in comparison. For natural gas processing plants, buffers are used to extract raster pixels from NLCD and the extracted pixels are reclassified so 21-Developed, Open Space, 22-Developed, Low Intensity, 23-Developed, Medium Intensity, 24-Developed, High Intensity, and 31-Barren Land are the same value, while all other classifiers are designated as having no data.

**(25) Natural Gas Storage Facilities**

For natural gas storage facilities buffers are used to extract raster pixels from NLCD and the extracted pixels are reclassified so 22-Developed, Low Intensity, 23-Developed, Medium Intensity, 24-Developed, High Intensity, and 31-Barren Land are the same value, while all other classifiers are designated as having no data.

**(78) Municipal Landfills with Gas**

Since there were such few points that lacked polygons, 17 polygons are drawn from point data and merged with OSM data for landfills with gas. Then the merged polygon data is ‘Extracted by Mask’ from NLCD, and reclassified as 21-Developed, Open Space, 22-Developed, Low Intensity, 23-Developed, Medium Intensity, 24-Developed, High Intensity, 31-Barren Land, and 41- Deciduous Forest.

**(81) Municipal Waste Plant,**

**(85) Wood Waste Plant,**

**(51-59) General Renewable Metal Processing Plants**

The points are put into ArcMap and their land use areas are manually digitized using the aerial imagery and OSM base maps. These drawn polygons were used to extract raster pixels from NLCD and the extract pixels are reclassified so every unique classifier has the same value.

**(23) Natural Gas/Petroleum Power Plant**

For natural gas and petroleum power plant, some of their points exactly overlapped one another. Therefore, a new subclass is created where the polygons are hand drawn in ArcMap using the aerial and OSM base maps as reference. This new subclass is called natural gas/petroleum power plant and has no OSM polygon equivalent. These drawn polygons are used to extract raster pixels from NLCD and the resulting pixels are reclassified so every unique classification has the same value. The final result is raster polygons. There are no OSM polygon equivalents with which to mosaic.

**Further Explanations**

1. **Drawing Polygons**

This process of drawing polygons of course has its own assumptions. If there are two points found at the same facility, the whole developed cohesive facility is divided in half and each half is enveloping its designated point. However, when making the division there is an attempt to divide it according to how the facility looks using the aerial and OSM base maps in ArcMap. For example, coal and petroleum power plants are distinguishable. Coal power plants tend to have smoke stacks, large piles of coal near the facilities, and have larger facilities in general. Petroleum power plants tend to have large areas housing cylindrical storage containers. In terms of the developed areas such as the plants themselves, that is where the division line is drawn since- other than coal plants usually having smoke stacks- there is no way to know what specific facility is for one subclass or the other.
